# Screening of suitable cationic dopants for solar absorber material CZTS/Se: A first principles study

**DOI:** 10.1038/s41598-019-52410-3

**Published:** 2019-11-05

**Authors:** M. V. Jyothirmai, Himanshu Saini, Noejung Park, Ranjit Thapa

**Affiliations:** 1 SRM Research Institute & Department of Physics and Nanotechnology, SRM Institute of Science and Technology, Kattankulathur, 603203 Tamil Nadu India; 2 Department of Physics, Ulsan National Institute of Science and Technology (UNIST), Ulsan, 689-798 South Korea; 3 Department of Physics, SRM University-AP, Amaravati, 522502 Andhra Pradesh India

**Keywords:** Solar cells, Solar cells

## Abstract

The earth abundant and non-toxic solar absorber material kesterite Cu_2_ZnSn(S/Se)_4_ has been studied to achieve high power conversion efficiency beyond various limitations, such as secondary phases, antisite defects, band gap adjustment and microstructure. To alleviate these hurdles, we employed screening based approach to find suitable cationic dopant that can promote the current density and the theoretical maximum upper limit of the energy conversion efficiency (P(%)) of CZTS/Se solar devices. For this task, the hybrid functional (Heyd, Scuseria and Ernzerhof, HSE06) were used to study the electronic and optical properties of cation (Al, Sb, Ga, Ba) doped CZTS/Se. Our in-depth investigation reveals that the Sb atom is suitable dopant of CZTS/CZTSe and also it has comparable bulk modulus as of pure material. The optical absorption coefficient of Sb doped CZTS/Se is considerably larger than the pure materials because of easy formation of visible range exciton due to the presence of defect state below the Fermi level, which leads to an increase in the current density and P(%). Our results demonstrate that the lower formation energy, preferable energy gap and excellent optical absorption of the Sb doped CZTS/Se make it potential component for relatively high efficient solar cells.

## Introduction

In the field of thin film based solar cells, the kesterite Cu_2_ZnSn(S/Se)_4_ (CZTS/Se) has attracted substantial attention as a next generation absorber materials owing to its favorable opto-electronic properties^1–3^. Besides the overall cost-effectiveness, the CZTS/Se has high absorption coefficient (10^4^ cm^−1^)^4,5^ and appreciable direct optical band gap (1–1.5 eV)^5–7^. The non-toxicity, sustainability and the richness of elements in the Earth’s crust are the extra added advantages of these materials. Nevertheless, the highest conversion efficiency achieved for CZTS/Se (9.4/11.6%)^8,9^ is still lower than that of CIGS (≈23%)^10^ thin-film solar cells. The low efficiency of CZTS/Se can be attributed to common occurrence of complex structural defects including competitive secondary phases (CuS/Se, Cu_2_S/Se, ZnS/Se, SnS/Se, Sn(S/Se)_2_, Cu_2_Sn(S/Se)_3_, etc.,). Further, the minority carrier lifetime of CZTS is about one or two orders of magnitude shorter than the CIGS materials^11^, which casts a serious obstacle that has to be overcome. It has been expected that, if secondary phases are minimized, the so-called band tailing problems will decrease substantially, and thus the minority carrier lifetime will be enhanced, which makes the efficiency gap between CIGS and kesterite to be lowered. Therefore, to boost the efficiency of CZTS/Se, it is necessary to avail the doping approach, which can also be utilized in controlling the intrinsic defect.

Doping of CZTS/Se materials may tune the crystallinity and microstructure of the thin films. Especially, Cu poor and Zn rich films are considered as the most efficient devices with low cation disorder during phase formation. The alteration of anionic ratio (S/Se) is an effective way to get appropriate microstructure and optimum band gap. But the difficulties in controlling the S/Se ratio during thermal annealing process which limits their practical usage. On the other hand, the cation substitution can improve the optoelectronic properties and also controls the stoichiometry to achieve appropriate crystal phase. The introduction of Cd by appropriate Zn/Cd ratio into CZTS thin films increased the power conversion efficiency from 5.30% to 9.24%^12^. In a similar perspective, Xiao *et al*.^13^ doped the magnetic ions to obtain a larger Seebeck coefficient, lower thermal and higher electrical conductivity. Zhao *et al*.^14^ studied the structural, electronic and optical properties of Na doped CZTS using the generalized gradient approximated (GGA) density functional plus the Hubbard U potential (GGA+U) and found that the occupation of interstitial sites are favorable. The CZTS thin films with Na substitution can enhance the device efficiency by improving the open circuit voltage (V_oc_) and fill factor^15^. More interestingly, the introduction of both Na and Sb into CZTS thin films not only improves the power conversion efficiency up to 5.7%, but also reduces the disorder present in specific lattice positions by interacting with Cu and Sn sites^16^. All these suggest that, doping of CZTS/Se with cations can improve the crystallinity and efficiency.

In previous studies, antimony (Sb) as a dopant is found to improve the efficiency of CIGS. The surface morphology and kinetics of grain growth were improved by the incorporation of Sb into CIGS absorber layer^17,18^, which may enhance film quality under low annealing temperatures. Therefore, it is important to understand the effect of Sb on CZTS/Se in order to improve the efficiency. More interestingly, it is anticipated that the incorporation of Sb into CZTS/Se may play an important role similar to that of CIGS. Recently, Zhang *et al*.^19^, performed quantum mechanical calculations on Sb doped CZTS and found that Sb prefers to substitute Sn atomic sites. The Sb 5s states form an isolated half-filled intermediate band at 0.5 eV above the valence band maximum, which may contribute to the increase of the photocurrent as well as the solar cell efficiency.

In the present work, a screening based approach is used to find an appropriate cationic dopant to improve the performance of CZTS/Se solar cells. Further, the fundamental effects of four dopants (Al, Ga, Ba, and Sb) were systematically investigated using electronic calculations. The calculated formation energies and bulk modulus reveal that the Sb atom is more favorable to dope in the CZTS/Se structure. Our results demonstrate that the effect of dopants near the gap will tailor the electronic properties, which help to increase the optical absorption at visible region. The current density and power density of doped and co-doped CZTS/Se are estimated and compare with the pure materials. Overall, tuning the energy gap and the consequent visible light response of the doped CZTS/Se materials is the focus of the current work.

## Results and Discussion

### Structural information, chemical potential, formation energy and doping configuration

CZTS and CZTSe have a kesterite structure with space group I$\bar{4}$ and obey Lew’s octet rule with eight electrons around each anion atom (S or Se). First, we fully relaxed CZTS/Se (2x2x1) supercells and the obtained lattice parameters are a = 10.94/11.53 Å and c = 10.93/11.51 Å for CZTS/Se, which agrees well with the experimental values^20^. To check the effect of doping on the electronic and photon absorption properties of CZTS/Se, we consider, Sb, Al, Ga and Ba atoms as impurity cations. We utilized the screening based approach to determine the possible doping sites in the host materials. Initially, we placed the dopants at all possible cationic sites and compared the relative formation energies (E_f_). This is a commonly used method to correlate the corresponding degree of possibility of doping into the host lattice. The structural optimizations of doped CZTS/Se (2x2x1) supercells were performed using PBE-PAW method. After optimization, the total energy of the systems are obtained through HSE06 calculations. To assert the relative stability of Al, Ga, Sb and Ba doped CZTS/Se, we computed the formation energy (E_f_), which is defined as follows:

1 $$\Delta {H}_{D,q}({E}_{{\rm{F}}},{\mu }_{{\rm{i}}})=[{E}_{D,q}-{E}_{{\rm{H}}}]+\sum _{i}{n}_{{\rm{i}}}{\mu }_{{\rm{i}}}+q({E}_{{\rm{F}}}+{\varepsilon }_{{\rm{VBM}}})$$

Where, E_D,q_ (defect) and E_H_ (host supercell) are the total DFT energies. The chemical potential of the element i is μ_i_ which is either removed (n_i_ > 0) or added (n_i_ < 0) from the host supercell. E_F_ and ε_VBM_ corresponding to Fermi energy and the valence band maximum of host crystal, respectively. To determine Eq.(1), we need a range of variables μ_i_  and E_f_. However, the presence of secondary phases makes it difficult to locate the allowed region for chemical potentials, μ_i_ . The Fermi energy E_f_ typically varies between conduction band minimum and valance band maximum. i.e., from 0 to E_g_. From Eq.(1), we can understand that the defect formation energy mainly depends on chemical potential, which is thermodynamically limited by several conditions

The Cu is highly preferable to form the pure FCC metal (i.e., μ_Cu_<=0), which suppresses the formation of single crystal CZTS/Se. In order to avoid this phase, it is required that μ_Cu_< 0, similarly μ_Zn_< 0, μ_Sn_< 0 and μ_S/Se_< 0. Subsequently, the stable CZTS/Se crystal structure can be maintained if the chemical potential of Cu, Zn, Sn and S/Se satisfies the following equations:

2 $$\begin{array}{l}2{\mu }_{{\rm{Cu}}}+{\mu }_{{\rm{Zn}}}+{\mu }_{{\rm{Sn}}}+4{\mu }_{{\rm{S}}}=\Delta {H}_{{f(\mathrm{Cu}}_{{\rm{2}}}{{\rm{ZnSnS}}}_{{\rm{4}}})}=-4.79eV\\ 2{\mu }_{{\rm{Cu}}}+{\mu }_{{\rm{Zn}}}+{\mu }_{{\rm{Sn}}}+4{\mu }_{{\rm{Se}}}=\Delta {H}_{{f(\mathrm{Cu}}_{{\rm{2}}}{{\rm{ZnSnSe}}}_{{\rm{4}}})}=-4.5eV\end{array}$$

where ΔH_f_(Cu_2_ZnSn(S/Se)_4_) is formation enthalpy. Further, to avoid the formation of secondary phases such as CuS, Cu_2_S, ZnS, SnS, SnS_2_, and Cu_2_SnS_3_ for which the formation energy is greater than the sum of the chemical potentials of the constituent elements of a competing phase. The relations for ΔH_f_(CuS/Se) ΔH_f_(Cu_2_S/Se), ΔH_f_(ZnS/Se), ΔH_f_(SnS/Se), ΔH_f_(Sn(S/Se)_2_) and ΔH_f_(Cu_2_Sn(S/Se)_3_) are given in the supplementary information. The considered secondary phases whose formation energies shown in Table S1 exhibits good agreement with experimental results.

**Figure 1:**
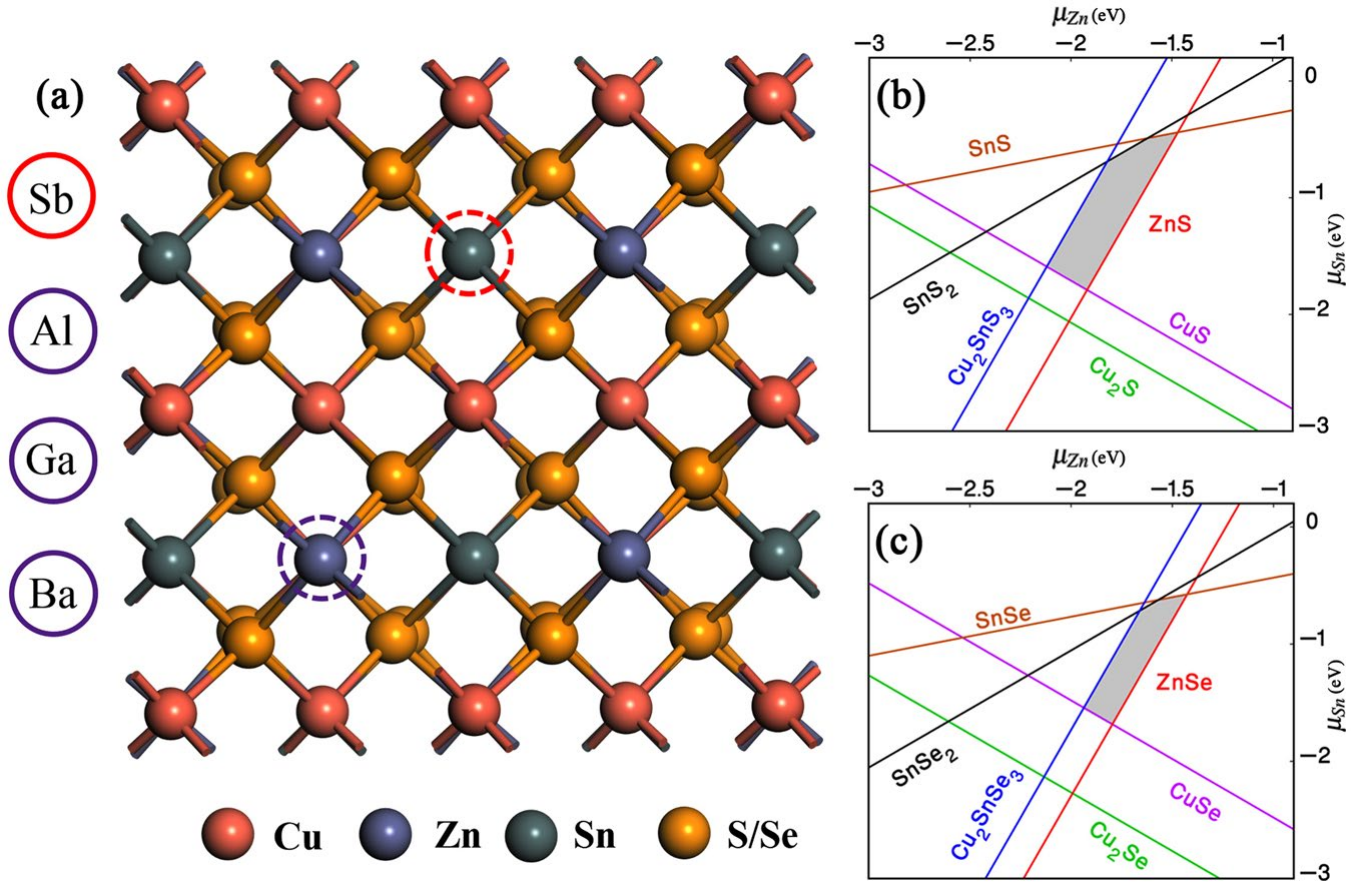
(**a**) The relaxed atomic structures of the pure and doped CZTS/Se, red and violet coloured dotted circles indicates the dopant sites of Sb and (Al, Ga, Ba) in place of Sn and Zn respectively, (**b**) and (**c**) The calculated stable region of phase diagram for pure CZTS and CZTSe as a function of Zn and Sn chemical potential with *μ*_Cu_ =-0.5 eV, estimated using HSE06 functional.

**Figure 2:**
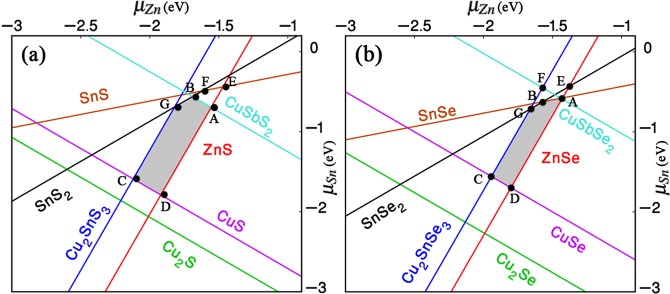
(**a**,**b**) The calculated stable region of phase diagram for Sb doped CZTS and CZTSe as a function of Zn and Sn chemical potential with *μ*_Cu_ = -0.5 eV, estimated using HSE06 functional. The shaded areaindicates the stable chemical potential region.

Theoretically, the stable quaternary phase relative to the secondary phases can be identified by analyzing the chemical potential space under equilibrium conditions, i.e., Cu-rich and Zn-poor conditions are unable to locate single phase kesterite structure (see Fig. S1) and produces a handful of secondary compounds. This is in contrast to the high efficiency devices associated with Cu-poor and Zn-rich growth. Therefore, it is necessary to develop non-equilibrium techniques (i.e., Cu-poor and Zn-rich conditions) to avoid the secondary phases. The chemical potential region is spanned to a polyhedron 3D space with three independent variables (μ_Cu_, μ_Zn_, μ_Sn_) for clear visualization, a part of the polyhedron is taken in the Δμ_Cu_=−0.5eV plane, as shown in Fig. 1b,c, the Δμ_Cu_ is in between 0 and −0.7 eV. The thin grey area represents the stable region of CZTS/Se against various secondary phases. More interestingly, the shapes of stable region for both CZTS and CZTSe are identical considering the presence of the secondary compounds. Further, the boundaries of the stable region is mostly shared by Cu_2_Sn(S/Se)_3_ phase on the left and ZnS/Se phase on the right, when Zn is too poor or too rich condition, respectively. This reveals that Cu_2_Sn(S/Se)_3_ and ZnS/Se phases grow more easily than other secondary compounds during the formation of stable CZTS/Se phase due to non-uniform control of chemical potentials. When Sb introduce to compound CZTS/Se secondary phase CuSb(S/Se)_2_ is considered i.e., the region around ABGCD (see Fig. 2a,b). The chemical potentials for each element at A-D are shown in Table S1, where it can be see that μ_Zn_ value becomes lower which indicates Zn poor condition when we are going towards A to B to C. While μ_Sn_ value becomes lower which indicates Sn poor condition when we are going towards B to C to D. For Sb related defect formation energy, we consider chemical potential at point A (Table S2) and for Al, Ba, Ga, we consider chemical potentials as pure metal phases which is the value of −4.26, −3.56, −2.32 respectively.

In the case of a single Al atom replaced the Zn atom of CZTS/Se (2 × 2 × 1) supercell, the defect formation energy is found to be about 1.01/0.95 eV. We use the same format to represent the values of CZTS/Se in the whole manuscript. Further, the defect formation energies for Al occupying Cu and Sn sites of CZTSe (2x2x1) supercell are 4.71/4.38 and 3.21/3.38 eV respectively. The values of formation energy are given in Table 1. On comparison of formation energy it can be revealed that Al occupying Zn site is more favorable, which can be considered for further study to estimate the electronic structure and photon conversion efficiency. Similarly we found that Sb_Sn_ (0.53/0.41 eV), Ga_Zn_ (2.07/1.71 eV), Ba_Zn_ (−0.14/−0.26 eV) are more suitable as dopant sites.

**Table 1:** Calculated lattice parameters, bond length (BD), bulk modulus (BM), formation energy (FE) and Bader charge (BC) of CZTS/Se.

Structure	Lattice parameters (Å)	BD (Å)	BM (GPa)	FE (eV)	BC (e)
	a	c			
Pure	10.94/11.53 10.93/11.51	Sn-2.47/2.62	65.80/53.00	–	*
		Zn-2.47/2.5			
Sb_Sn_	10.95/11.52 10.95/11.55	2.57/2.71	65.00/51.25	0.53/0.41	1.27/1.0
Al_Zn_	10.95/11.54 10.94/11.54	2.30/2.45	65.69/52.83	1.01/0.95	2.11/1.98
Ga_Zn_	10.97/11.54 10.94/11.56	2.36/2.49	65.39/48.83	2.07/1.71	1.03/0.81
Ba_Zn_	11.00/11.60 11.09/11.66	2.93/3.1	58.68/49.23	−0.14/−0.26	1.41/1.39

The values corresponding to various properties are projected in the order of CZTS/CZTSe. *See the charge analysis section for detail information.

Secondly, we also estimate the bulk modulus of the systems to check the mechanical stability of the doped CZTS/Se structures. It is an important physical parameter of crystals; that reflects bonding characters in crystals and, in many instances, is used as an indicator for crystal strength and hardness^21–27^. The third order birch murnaghan equation of state is used to compute the bulk modulus through fitting the total energies with cell volumes of the material. The pure CZTS/Se (2x2x1) supercell is found to have a bulk modulus of about 65.8/53 GPa. Whereas, the bulk modulus of Al occupying Cu, Zn and Sn sites of CZTSe are 51.93 GPa, 52.83 GPa and 53.6 GPa, respectively. The bulk modulus of Al placed in Zn site (which is the favorable site of doping) is similar than pure CZTSe value, indicate the good stability of the doped system. For the case of Sb as dopant, Sn is the most favorable site^19^ and the bulk modulus is 65/51.25 GPa, which is comparable to pure case. In the case of Ga_Zn_, Ba_Zn_ CZTS/Se systems, the bulk modulus are 65.39/48.83, 58.68/49.23 GPa respectively shows lower stability compare to pure. The bulk modulus values of pure and doped system are tabulated in Tables 1 and S1 of the supplementary information. It can be seen that Sb leads to a suitable dopant, considering both formation energy and bulk modulus.

The graphical illustration of doped CZTS/Se is shown in the Fig. 1a and the value of lattice parameter of the pure and doped supercells and bond distance between impurity cation-anion is shown in Table 1. The lattice parameters of Sb, Al, Ga doped CZTS/Se are almost similar to the pure materials, whereas in the case of Ba as dopant, the host lattice parameter increase by about 0.6%. After placing the impurities in their respective positions, the atoms nearer to the dopants will tend to displace themselves to find new equilibrium positions. For Sb doped CZTS/Se, the computed bond length between Sb and S/Se is about 0.1/0.09 Å longer than that of corresponding Sn-S/Se bonds in pure CZTS/Se, which are 2.47/2.62 Å, respectively. In the case of Al doped CZTS/Se, the Al-S/Se bond length is around 0.06/0.05 Å shorter than that of Zn-S (2.47 Å)/Se (2.5 Å) bonds in pure CZTS/Se. In Ba doped CZTS/Se, the Ba-S/Se bond length is 0.57/0.6 Å longer than the pure material. The Ga doped CZTS/Se show similar bond lengths compared to host CZTS/Se. From the above analysis, the larger dopant atom tends to push the neighbouring atoms outwards, whereas, the smaller dopant atom pulls the surrounding atoms inward.

Further, we calculated the bond strength between cation and anion by considering various models to check the effect of doping on the local stability. For this purpose, we consider the Se vacancy which is bonded with Sn or Zn atom or with the corresponding dopant atoms. The formation energy of Se vacancy values are presented in Table S3 as defined by the following equation.

3 $${E}_{f}({V}_{Se})={E}_{t}^{V}(defect)-{E}_{t}(H)+{E}_{t}(Se)$$

Where ${E}_{t}^{V}(defect)$ is the total energy of the supercell with a vacancy and *E*_*t*_(*H*) is the total energy of the host crystal, *E*_*t*_(*Se*) is the energy of single Se atom considering its bulk form (trigonal, P3121) respectively.

The formation energy of Se vacancy for Ba doped CZTSe is estimated to be 0.36 eV, which indicates very less bond strength between Ba-Se. Even though the formation energy of Ba occupying Zn site is minimal (−0.26 eV), the less binding of Ba and Se (or easy formation of Se vacancy) lead the structure unstable. In the case of Al and Ga as dopants, 2.1 eV and 1.58 eV is the binding strength between Al-Se and Ga-Se bonds (or Se vacancy formation energy). These bonds are quite strong compared to other dopants, but the corresponding defect formation energies for Al and Ga occupying Zn sites are very high. We can see that in case of Sb doped CZTSe, the formation energy for Se vacancy (or Sb-Se bond strength) is 1.26 eV. The formation energy of Se vacancy in pure and Sb, Al, Ga, Ba doped CZTSe are tabulated in Table S3 of the supplementary information. From the bulk modulus and the formation energy of anion (Se) vacancy, we can easily infer that Ba is quite an unstable dopant for CZTS/Se.

We performed Bader charge analysis to describe the variations in atomic charges as a consequence of doping. For pure CZTS/Se, the Zn, Sn, S/Se atoms have Bader charges of +0.8/+0.67e, +1.39/+1.11e, −0.76/−0.64e, respectively. When Al atom is injected into the system, the Bader charge of Al atom for CZTS/Se (+2.11/+1.98e) is found to increase than the replaced Zn atom of pure CZTS/Se. This indicates that the S/Se atoms are pulled towards the dopant due to a reduction in the Coulomb repulsion, which is inconsistent with the above mentioned Al-S/Se bond lengths. The similar behavior is found in Ga_Zn_ (+1.03/+0.81e), Ba_Zn_ (+1.41/1.39e) dopants, where the Bader charge is increased than the substituted atoms of pure systems. The Bader charge of Sb atom for CZTS/Se is +1.27/+1.0e.

### Electronic properties

**Figure 3:**
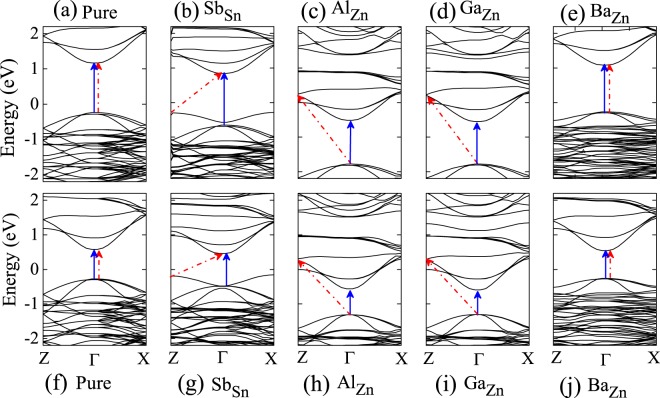
The band structures of the pure CZTS/Se and the Sb_Sn_, Al_Zn_, Ga_Zn_, Ba_Zn_ doped CZTS/Se, top layer shows for CZTS and bottom layer shows for CZTSe. Blue & red color arrow indicates bulk energy bandgap (E_g_) & dopant-bulk energy gap (E_I_), Fermi level is at 0 eV.

Incorporating impurity atom into the materials to tailor their electronic structures have been widely implemented as an effective strategy. Especially the bulk energy band gap (E_g_), separating the valence and conduction bands would dramatically influence the conversion efficiency of solar cells. Here, we introduce dopant-bulk energy gap, denoted by E_I_, which corresponds to the energy difference between the dopant-derived energy band and the bulk valence/conduction band. In order to corroborate how dopants reshape the electronic properties of CZTS/Se, the electronic band structures of doped and undoped CZTS/Se are presented in the Fig. 3. Blue line indicates E_g_ and red line indicates E_I_. These intermediate bands can be effectively analysed by their charge transition levels, as they belong to particular values for which two charge states of chemical potential have same formation energies. This plays a crucial role when the chemical potential is not well specified, especially during the complex process of CZTS/Se. For pure CZTS/Se, the valence band maximum (VBM) and conduction band minimum (CBM) are placed at the same Γ point, leading to a direct band gap transition. The band gaps are found to be 1.43 eV and 0.85 eV for CZTS and CZTSe, respectively, which is shown in Fig. 3a,f agrees well with the previous experimental and theoretical values. After doping, the electronic structures show significant variations in the pattern of energy bands, as a ramification of structural changes. For Sb doped CZTS Fig. 3b, a new intermediate band is created just above the VBM, while CBM remains unchanged. Thus the dopant-bulk energy gap is 1.14 eV due to the creation of a defect state. The similar behavior is also observed for Sb doped CZTSe, where the E_*I*_ is 0.6 eV. The observed intermediate bands play a great significance to separate and transfer the electrons from one state to other states. It is also noteworthy that the Sb dopant has shifted the electron transition from direct to indirect. For a better explanation, we also calculated the projected density of states to locate the exact position of energy levels related to impurity states (See Figs S3 and S4). For the case of Al and Ga doped CZTS/Se, the impurity levels overlap with the CB, so the first CZTS/Se resulting in higher energy gaps, thus the dopant-bulk energy gap is 1.97/1.55 eV, 1.98/1.56 eV. Further, the energy bands are found to shift towards the lower energy levels. On the other hand, the band structure of Ba doped CZTS/Se remains same as pure compounds with similar band gaps (1.36/0.8 eV). It is also notable that the Sb and Ba dopants show lower gaps, whereas, Al and Ga impurities possess much higher gaps compared to pure systems. Further, we also calculated the electronic structure by increasing the size of supercell to 2 × 2 × 2, which contains 128 atoms. To overcome the computational cost of Hybrid functions, we implemented Hubbard U (PBE+U) correction method^28^. The Coulomb interaction (U) of 8.5 eV and 5.5 eV is considered for Cu 3d and Sn 4d orbitals respectively. The band structure of pure and doped CZTS/Se using Hubbard U correction is shown in the Fig S10. For pure CZTS/Se, the VBM and CBM are placed at the same Γ point, leading to a direct band gap transition. The band gaps are found to be 0.96 eV and 0.39 eV for CZTS and CZTSe, respectively. When compared with HSE band structure, the band gap of PBE+U structure is mostly reduced, whereas the whole band dispersion is similar for both Hubbard U and hybrid functional method. Hence, even though we reduced the solubility concentration, there is no significant difference in the band dispersion, defect level and our proposed science remains the same.

**Figure 4:**
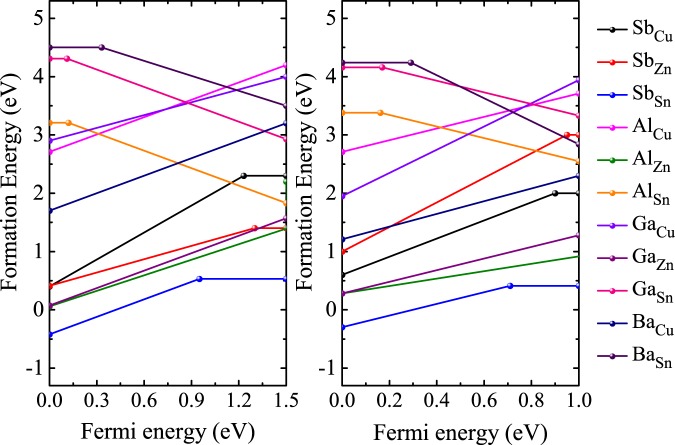
Calculated formation energies of intrinsic defects in doped CZTS and CZTSe as a function of Fermi energy (E_f_) at the chemical potential points A.

The formation energies not only depend on the atomic chemical potentials but also depend on the Fermi energy, according to Eq. (1). Typically, the defects play a major role in photovoltaic and carrier transport properties of thin film solar cells. It often produces levels near the band edge or in the region band gap. To study the intrinsic defect properties in CZTS/Se, we employed a well known correction method based on Lany and Zunger model^29^ and calculated the defect formation energy as a function of Fermi energy (E_*f*_) considering the values of chemical potential of point A (see the chemical potential region) as shown in the Fig. 2, where the slope of the line represents the charge state of a defect. Here the positive (negative) slope of the line indicates that the defect is a donor (acceptor) and can donate (accept) electrons to get ionized to a positively (negatively) charged state. The defect is said to be neutral (not ionized) if the slope is equal to zero. Further, the ionization energy levels in the band gap can be located through the cross points of the lines. From Fig. 4, the slope of the defect formation energies varies for differently charged defects as a function of Fermi energy. In both CZTS and CZTSe cases, it can be easily identified that the donor defect Sb_*Sn*_ has the lowest formation energy in almost the whole range of band gap and is significantly stabilized. It is also noteworthy that the formation energy of Sb_*Sn*_ is comparatively much lower than the generally observed Cu_*Zn*_ defect^30,31^. Moreover, the Sb_*Sn*_ defect can make a prominent contribution to n-type conductivity in CZTS/Se and is also consistent with the experimental observation^16,32^. Among all the defects, Al_*Zn*_ and Ga_*Zn*_ (CZTS/Se) has the next lowest formation energy of 0.07 eV and 0.28 eV, respectively, when Fermi energy approaches 0 eV.

**Figure 5:**
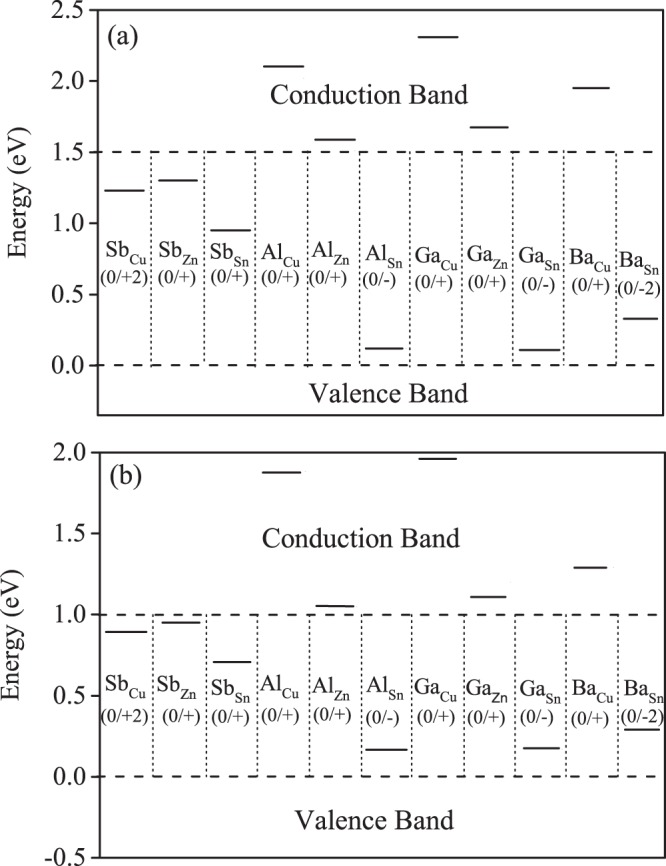
Calculated charge-state transition levels of doped (**a**) CZTS and (**b**) CZTSe.

Apart from the formation energies of the intrinsic defects, the charge-state transition levels of CZTS/Se can offer an effective way of exploring the photovoltaic properties. It is defined as the Fermi level where the defect state can accept or donate electrons. Figure 5 shows the calculated transition levels for all the studied defects in CZTS/Se. For CZTSe, Most of the intrinsic defects act as a donor defects except Al_*Sn*_, Ga_*Sn*_ and Ba_*Sn*_, which are acceptors close to the VBM level with the (0/-), (0/-) and (0/−2) transitions. Moreover, three donor defects (Sb_*Sn*_, Al_*Zn*_, Ga_*Zn*_) show significantly small defect formation energy and the remaining defects possess high defect formation energies, hence are not prominent for charge compensation. The Sb_*Sn*_ has the lowest formation energy and acts as a donor with the (0/+) transition at 0.71 eV, which prominently contributes to n-type conduction in CZTSe. The transition levels of Al_*Cu*_, Al_*Zn*_, Ga_*Cu*_, Ga_*Zn*_ and Ba_*Cu*_ are not within the bandgap. In the case of CZTS, Sb_*Sn*_ has the lowest formation energy with (0/+) a transition at 0.95 eV. Even Ba_*Sn*_ has the low defect formation energy and can act as a deep acceptor with transition (0/-) at 0.33 eV. The Al_*Sn*_ and Ga_*Sn*_ show shallow transition energy level located near the VBM. Similar to CZTSe, the Al_*Cu*_, Al_*Zn*_, Ga_*Cu*_ and Ga_*Zn*_ transition levels are outside the Fermi energy range. Overall, Sb_*Sn*_ show the lowest formation energy than any other studied dopants in both CZTS and CZTSe systems, thus the doping of Sb in high concentration at Sn atomic site can illuminate the future CZTS/Se based solar technology.

### Optical properties: current density and theoretical efficiency

**Figure 6:**
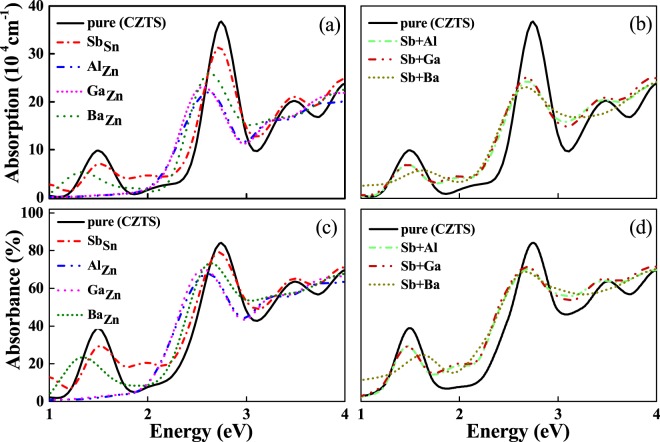
(**a**,**b**) Demonstrate the calculated absorption coefficients for Sb, Al, Ga, Ba-doped & codoped CZTS and (**c**,**d**) shows the absorbance (%) for pure and Sb, Al, Ga, Ba-doped and codoped CZTS.

**Figure 7:**
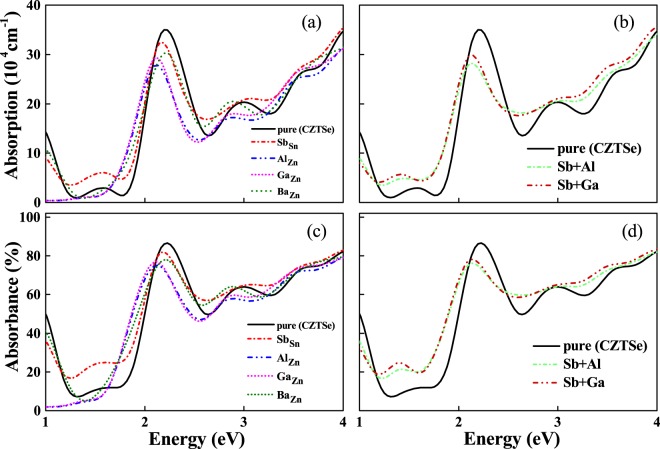
(**a**,**b**) Demonstrate the calculated absorption coefficients for Sb, Al, Ga, Ba-doped & codoped CZTSe and (**c**,**d**) shows the absorbance (%) for pure and Sb, Al, Ga, Ba-doped and codoped CZTSe.

The optical absorption spectrum plays a crucial role in solar cell materials by providing a better understanding about optimum solar energy conversion efficiency. It elucidates the penetration of light intensity at a particular energy (wavelength) into the substance before it get absorbed. To evaluate the influence of dopants on optical properties of CZTS/Se, we have computed the optical absorption coefficients of doped CZTS/Se and compared with the host materials as shown in the Figs 6 and 7. In order to get the absorption coefficient, we first calculated the extinction coefficient k through real (*ε*_1_) and imaginary (*ε*_2_) parts of the dielectric function using independent particle approximation and HSE06 functional.

4 $$\kappa (E)=\frac{1}{\sqrt{2}}\sqrt{-{\varepsilon }_{1}(E)+\sqrt{{\varepsilon }_{1}^{2}(E)+{\varepsilon }_{2}^{2}(E)}}$$

Where E represents the photon energy. Thus, the absorption coefficient denoted as *α*(E) can be written as

5 $$\alpha (E)=\frac{4\pi \kappa }{hc/E}$$

Figures 6 and 7 shows the absorption coefficient and absorbance (%) as a function of photon energy for doped and pure CZTS/Se. It can be seen that the pure CZTS show two major absorption peaks at 1.5 eV and 2.8 eV. In contrast, CZTSe contains only one absorption peak in the studied range at about 2.2 eV. We note that the obvious absorption peaks of CZTSe is shifted towards lower energies (ie., towards visible range) compared to CZTS and thus play a predominant role in the absorption enhancement. The effect of doping show marked variations in the peak positions and leads to a significant effect in tuning the optical performance of host materials. For Sb doped CZTS, an additional absorption peak is appeared in the visible range at around 1.8 eV along with the two major peaks at 1.5 eV and 2.8 eV. Whereas, for Sb doped CZTSe, the absorption peak in the visible light range is significantly enhanced and thus can be used to harvest much more solar energy compared to Sb doped CZTS. It is also note worthy that the absorption energy region is broadened in both the cases due to the presence of intermediate levels just above the VBM. This is related to the electron transition from the intermediate band to CB. Therefore, Sb doped CZTS show a two step optical transition across valance and conduction bands through intermediate bands, thus leading to extended absorption energy range with enhanced light absorption capacity. On the other hand, Al and Ga dopants show only one absorption peak in the high energy region at around 2.5 eV for CZTS and 2.2 eV for CZTSe and no absorption is found in the energy range below 2.0 eV. The absence of absorption in the visible light range clearly demonstrates that the Al and Ga dopants cannot harvest solar energy. Even though the substitution of Ba in CZTS/Se materials show some shift in the energy range, but could not able to provide any significate improvement in the visible light region. Therefore, these calculations demonstrate that among all substitutes, Sb doped CZTS/Se show marked enhancement in the visible light absorption and thus can be used to harvest solar energy. Further, the possible effectiveness of co-doped CZTS/Se also qualitatively analysed to understand their role in improving the photovoltaic efficiency. Since, Sb substituent possess remarkably high performance than the other studied dopants, therefore, in all cases, we used Sb as one of the co-dopant while changing the other atoms (Al, Ga and Ba). There are three notable aspects for the codoped structures: First one is related to their peak intensity, where the strength of the peaks are suppressed in comparison with the host materials. Secondly, the optical absorption is shifted towards lower energies. Thirdly, an additional absorption peak with pronounced intensity is observed at around 1.8 eV for CZTS and 1.5 eV for CZTSe doped structures. These results illustrate that the substitution of Sb (both single and codoped) into CZTS/se materials can improve the sunlight absorption and enable the use of Sb doped CZTS/Se in more efficient photovoltaic devices.

For better comparison with other dopants, we have calculated integral value of absorbance in the two ranges. 380–780 nm (visible) and above 780 nm (>infrared) and the values are presented in the Table S6. The main difference can be seen in the visible region absorbance. The integral value for Sb doped CZTSe in the visible limit increases by about 12% compared to the pure compound, and the corresponding current density is enhanced by 16% in total. In case of other dopants (Al, Ga and Ba), visible region absorbance is lower than the pure CZTS/Se, except for the visible limit of Ba doped CZTS system which is 0.6% higher than pure structure and the corresponding current densities are also fall in the same range, indicating that the importance of visible region absorbance in enhancing the current density. The integral value of absorbance for Sb doped CZTS/CZTSe is showing higher value in visible and IR range compared to pure and other element doped CZTS/Se, indicating the cause for higher current density.

Further we have calculated refractive index (n) by using extinction coefficient *κ*

6 $$n(E)=\frac{1}{\sqrt{2}}\sqrt{{\varepsilon }_{1}(E)+\sqrt{{\varepsilon }_{1}^{2}(E)+{\varepsilon }_{2}^{2}(E)}}$$

and the reflectivity were calculated by using n and *κ*

7 $$R(E)=\frac{{(n-\mathrm{1)}}^{2}+{\kappa }^{2}}{{(n+\mathrm{1)}}^{2}+{\kappa }^{2}}$$

The refractive index and reflectivity as a function of photon energy (eV) for pure and doped CZTS/Se are shown in the Figs. S11, S12. For pure CZTS/Se, the static refractive index (*n*_0_) is found to be 2.58/3.2. For Sb doped CZTS/Se, the refractive index values increases to 2.66/3.34 and for Ba doped CZTS/Se the values are 2.6/3.25, respectively. Whereas Al doping into CZTS/Se, a decrease in the refractive index to 2.5/2.73 is observed. The same decreasing trend is observed for Ga doped CZTS/Se with a refractive index of 2.5/2.75. For co-doped CZTS/Se structures, the refractive index of Sb+Al, Sb+Ga and Sb+Ba are found to be 2.62/3.14, 2.63/3.2 and 2.68/3.39, respectively. The reflectivity as a function of photon energy for all the cases shows similar trend as of refractive index. Further, the refractive index reaches its maximum value of 2.88/3.8 at 1.2/0.66 eV for pure, 2.79/3.68 at 0.85/0.42 eV for Sb doped CZTS/Se, 3.09/3.5 at 2.23/1.84 eV for Al doped CZTS/Se, 3.13/3.58 at 2.23/1.84 eV for Ga doped CZTS/Se and 2.99/3.83 at 1.24/0.56 eV for Ba doped CZTS/Se. The relatively high refractive index of Sb doped CZTS/Se materials make it suitable for absorber layer.

The absorbed photon flux J_abs_ under Air Mass 1.5G solar illumination is estimated using following equation.

8 $${J}_{abs}=e{\int }_{{E}_{g}}^{\infty }A(E){J}_{ph}(E)dE$$

where E_*g*_ is the band gap, E is the photon energy, A is the Absorbance and J_*ph*_ is the incident photon flux (units of photons/cm^2^.s.eV). The absorbed photon flux can be expressed as the equivalent short-circuit current density (J_sc_) mA/cm^2^ when we are multiplying with elementary charge e^33^.

The theoretical upper limit of the energy conversion efficiency P is depend on the overlap between the absorbance and the solar spectrum:

9 $$P=\frac{{\int }_{0}^{{\lambda }_{max}}W(\lambda )A(\lambda )C(\lambda )d\lambda }{{\int }_{0}^{\infty }W(\lambda )d\lambda }$$

Where W(*λ*) is the solar spectral irradiance at AM 1.5G. The *λ* and *λ*_*max*_ are the photon wavelength and the longest wavelength that can be absorbed by a material, respectively, which is obtained via electronic band gap E_*g*_:

10 $${\lambda }_{max}=\frac{hc}{{E}_{g}}$$

The absorbance A(*λ*) and the conversion factor C(*λ*) are given by:

11 $$A(\lambda )=1-{e}^{-\alpha (\lambda )d}$$

12 $$C(\lambda )=\lambda \frac{{E}_{g}}{hc}$$

Where *α*, d and E_*g*_ represents the absorption coefficient, thickness and the minimum band gap of the material, respectively.

The calculated dopant-bulk energy gap, current density and upper limit of the energy conversion efficiency P (%) of pure CZTS is found to be 1.43 eV, 20.01 mA/cm^2^ and 13.12% respectively, whereas the pure CZTSe possess dopant-bulk energy gap of 0.85 eV, current density about 35.08 mA/cm^2^ and P is 25.87%, (for doped systems see Tables 2 and 3). It is clear that the pure CZTSe possess significantly high P (%) compared to CZTS. This can be correlated with the corresponding dopant-bulk energy gaps, where the reduction in the gap enhances the photocurrent density and energy conversion efficiency. More interestingly, doping show drastic variations in both the current density and power conversion efficiency. The photocurrent density of Sb doped CZTS (25.02 mA/cm^2^) is incredibly 25.03% higher than the host material, while 5.84 mA/cm^2^ increment in current density is found for Sb doped CZTSe. This indicates that additional excited photons were produced from VB to CB. The corresponding P (%) for Sb doped CZTS (P = 17.47%) show exceptionally 33.2% higher than the pure CZTS, while 26.3% enhancement is observed for Sb doped CZTSe compare to pure one. The impurity state needs to satisfy some stipulations to enhance the conversion efficiency, which can be found in other reported works^34,35^. When Al and Ga are doped into CZTS, clear reductions of current density by 3.21 and 2.98 mA/cm^2^ can be seen. Also the power conversion performance for both the doped structures dropped by 2.44%. Similar adverse results were observed for the case of Al and Ga doped CZTSe, where the current density suppresses to a greater extent from 35.08 mA/cm2 to 29.65 and 30.07 mA/cm^2^ and thus the P (%) is reduced to 18.83% and 19.1%, respectively. It is noteworthy that, on the other hand, the Ba substituent does not show any difference in the photovoltaic energy conversion efficiency.

**Table 2:** Calculated dopant-bulk energy gap, current density and upper limit of the energy conversion efficiency P (%) of pure and doped CZTS considering 50nm film thickness.

system	dopant-bulk energy gap (eV)	current density (mA/cm^2^)	P %
Pure	1.43	20.01	12.12
Sb_Sn_	1.14	25.02	17.47
Al_Zn_	1.97	16.80	09.68
Ga_Zn_	1.94	17.03	09.82
Ba_Zn_	1.36	20.60	13.21
Sb+Al	0.92	24.90	17.45
Sb+Ga	0.86	25.60	18.06
Sb+Ba	1.27	24.3	16.42

**Table 3:** Calculated dopant-bulk energy gap, current density and upper limit of the energy conversion efficiency P (%) of pure and doped CZTSe considering 50 nm film thickness.

system	dopant-bulk energy gap (eV)	current density (mA/cm^2^)	P %
Pure	0.85	35.08	25.87
Sb_Sn_	0.6	40.92	32.68
Al_Zn_	1.55	29.65	18.83
Ga_Zn_	1.56	30.07	19.10
Ba_Zn_	0.8	37.56	28.23
Sb+Al	0.42	40.97	32.48
Sb+Ga	0.37	41.74	33.27
Sb+Ba	0.7	42.68	35.76

As shown in the Tables 2 and 3, the dopant-bulk energy gap of co-doped materials is relatively lower than the single doped CZTS/Se materials. Furthermore, the dopant-bulk energy gap of Sb and Ga codoped CZTS (0.86 eV) is much smaller and thus possess highest current density (25.60 mA/cm^2^) and energy conversion efficiency (18.06%) than the other co-dpants. On the other hand, the Sb and Ba co-doped CZTSe possess highest current density (42.68 mA/cm^2^) and P% (35.76%) than the other codopants. Since, Sb dopant only creates impurity levels, there is no much difference in the performance of co-dopants compared to single Sb doped CZTS/Se materials. Overall, these analysis confirm that the presence of impurity state and the narrow dopant-bulk energy gap by doping enhances the photo current density and upper limit of the energy conversion efficiency of CZTS/Se. A brief experimental report on the electrical performance parameters of doped and undoped CZTS/Se materials are summarized in Table 4. The CZTSSe device is shown to exhibit the best Power Conversion Efficiency (PCE) of 12.6% which is estimated by using V_oc_ and J_sc_. As per the authors survey, there are no theoretical calculations predicting the upper limit energy conversion efficiency of CZTS/Se. Hence we compare our estimated maximum energy conversion efficiency with experimental PCE to guide and suggest that the Sb as dopant should be tried by experimentalist broader manner to achieve the higher efficiency.

**Table 4:** Electrical performance parameters (open circuit voltage (V_*oc*_), short circuit current density (J_*sc*_), fill factor (FF) and conversion efficiency (*η*)) of CZTS/Se considering only experimental work.

System	V_*oc*_ (mV)	J_*sc*_ (mA/cm^2^)	FF(%)	*η*(%)
CZTS^8^	700	21.3	63	9.4
CZTSe^9^	423	40.6	67.3	11.6
CZTSSe^36^	513.4	35.2	69.8	12.6
CZCTS^12^	581	24.1	66	9.24
CZTS-Sb^16^	563	15.3	58.8	5.1
CZTS-Sb+Na^16^	610	14.9	63	5.7
CZTSe-J-Na^37^	409	35	54	7.9
CZTSe-J-Na+K^37^	421	36.2	53	8.3
CZTSSe-Ge^38^	403	28.51	46.83	5.38
Cu_1.8_Ag_0.2_ZnSnSe_4_^39^	298	44	26	3.4
Mo/Ag/CZTS^40^	596	14.38	41	3.51
CZGSe^41^	744	16	46	5.5
CZTSSe^42^	476	29	59	7.4
CIGS^43^	722	39.4	78.2	22.3

## Conclusions

In summary, we have presented the first-principles calculations to screen and examine the current density and upper limit of the energy conversion efficiency of CZTS/Se materials through cation (Al, Sb, Ga, Ba) substitution. The obtained results show that the formation energy of Al and Ga are relatively higher, whereas Sb and Ba possess lower formation energies. Further, the bulk modulus of Sb substituent (65/51.25 GPa) with pure CZTS/Se (65.8/53 GPa) confirms it as most suitable dopant. The electronic and optical properties of doped CZTS/Se have uncovered a few microscopic ingredients that result in different performances than pure materials. The Sb doped CZTS/Se show major variations in the electronic structure by incorporating an intermediate band in the dopant-bulk energy gap. More interestingly, the influence of doping decreases the dopant-bulk energy gap and improves the photocurrent density and upper limit of the energy conversion efficiency. The Sb doped CZTSe lifted the solar energy harvesting and accomplished an improvement in the energy conversion efficiency from 25.81% (pure CZTSe) to 32.68% with 26.3% enhancement.

### Computational details

We employ projector augmented wave (PAW) method^44^ for all the calculations via Vienna Ab initio Simulation Package (VASP)^45^. The exchange-correlation energy is introduced by generalized gradient approximation (GGA) as parametrized by Perdew, Burke, and Ernzerhof (PBE)^46^ for cell relaxation only. The conjugate-gradient algorithm is used to optimize the structures with the energy convergence criterion of 10^−5^ eV and k-point mesh of 7 × 7 × 7 is used. Relaxation was carried out by maximum forces on each atom are less than 0.01 eV/Å. It has been well documented that, for 3d or more correlated electrons, the GGA functional often result in poor results such as collapse of band gap of Mott insulator or erroneous overlap between d-electron band and p-electron band. By keep this in mind, in the present work, we implemented Heyd Scuseria Ernzerhof 06 (HSE06)^47^ hybrid functionals, which is more accurate than standard DFT-GGA functional for calculating the band structures, absorption coefficient and energetics. We consider the supercell approach to neglect the interactions with neighbouring cells of defect molecules. The Γ -centered k-point mesh of 2 × 2 × 2 is used for Brillouin zone integration.

## Supplementary information

Supplementary Information is available for this paper at https://doi.org/10.1038/s41598-019-52410-3.
