# Supplementary material for: Screening of suitable cationic dopants for solar absorber material CZTS/Se: A first principles study
Source: Sci Rep. 2019 Nov 5;9:15983. doi: 10.1038/s41598-019-52410-3 (PMC6831578; doi:10.1038/s41598-019-52410-3)
Supplement: Supplementary file 1 — Supplementary Information is available for this paper at https://doi.org/10.1038/s41598-019-52410-3. [file 41598_2019_52410_MOESM1_ESM.docx]

Supplementary information:

**Screening of Suitable Cationic Dopants for Solar Absorber Material CZTS/Se: A first principles study**

M. V. Jyothirmai*, Himanshu Saini*, Noejung Park^†^ and Ranjit Thapa**^,#^*^‡^

***SRM Institute of Science and Technology, Kattankulathur 603203, Tamil Nadu, India.

^#^Department of Physics, SRM University-AP, Amaravati 522502, Andhra Pradesh, India.

^†^Department of Physics, Ulsan National Institute of Science and Technology (UNIST), Ulsan 689-798, South Korea, Ulsan 689-798, Republic of Korea.

^‡^ranjit.t@srmap.edu.in

To avoid the formation of secondary phases such as CuS/Se, Cu_2_S/Se, ZnS/Se, SnS/Se, Sn(S/Se)_2_, and Cu_2_Sn(S/Se)_3_, the following relations should be satisfied.

For CZTS

$$\Delta\mu_{\mathrm{Cu}}+\Delta\mu_{S}<\Delta H_{f}\left( CuS \right)=-0.52 eV$$

$$2\Delta\mu_{\mathrm{Cu}}+\Delta\mu_{S}<\Delta H_{f}\left( Cu_{2}S \right)=-0.93 eV$$

$$\Delta\mu_{\mathrm{Zn}}+\Delta\mu_{S}<\Delta H_{f}\left( ZnS \right)=-1.94 eV$$

$$\Delta\mu_{\mathrm{Sn}}+\Delta\mu_{S}<\Delta H_{f}\left( SnS \right)=-0.91 eV$$

$$\Delta\mu_{\mathrm{Sn}}+2\Delta\mu_{S}<\Delta H_{f}\left( SnS_{2} \right)=-1.33 eV$$

$$2\Delta\mu_{\mathrm{Cu}}+\Delta\mu_{\mathrm{Sn}}+3\Delta\mu_{S}<\Delta H_{f}\left( Cu_{2}SnS_{3} \right)=-2.65 eV$$

$$\Delta\mu_{\mathrm{Cu}}+\Delta\mu_{\mathrm{Sb}}+2\Delta\mu_{S}<\Delta H_{f}\left( CuSbS_{2} \right)=-1.27 eV$$

For CZTSe

$$\Delta\mu_{\mathrm{Cu}}+\Delta\mu_{\mathrm{Se}}<\Delta H_{f}\left( CuSe \right)=-0.48 eV$$

$$2\Delta\mu_{\mathrm{Cu}}+\Delta\mu_{\mathrm{Se}}<\Delta H_{f}\left( Cu_{2}Se \right)=-0.81 eV$$

$$\Delta\mu_{\mathrm{Zn}}+\Delta\mu_{\mathrm{Se}}<\Delta H_{f}\left( ZnSe \right)=-1.8 eV$$

$$\Delta\mu_{\mathrm{Sn}}+\Delta\mu_{\mathrm{Se}}<\Delta H_{f}\left( SnSe \right)=-0.95 eV$$

$$\Delta\mu_{\mathrm{Sn}}+2\Delta\mu_{\mathrm{Se}}<\Delta H_{f}\left( Sn{Se}_{2} \right)=-1.28 eV$$

$$2\Delta\mu_{\mathrm{Cu}}+\Delta\mu_{\mathrm{Sn}}+3\Delta\mu_{\mathrm{Se}}<\Delta H_{f}\left( Cu_{2}Sn{Se}_{3} \right)=-2.56 eV$$

$$\Delta\mu_{\mathrm{Cu}}+\Delta\mu_{\mathrm{Sb}}+2\Delta\mu_{\mathrm{Se}}<\Delta H_{f}\left( CuSb{Se}_{2} \right)=-1.24 eV$$

Where $\Delta H_{f}\left( CuS/Se \right)$, $\Delta H_{f}\left( Cu_{2}S/Se \right)$, $\Delta H_{f}\left( ZnS/Se \right)$, $\Delta H_{f}\left( SnS/Se \right)$, $\Delta H_{f}\left( Sn(S/{Se)}_{2} \right)$, $\Delta H_{f}\left( Cu_{2}Sn(S/{Se)}_{3} \right)$, $\Delta H_{f}\left( CuSb(S/{Se)}_{2} \right)$ are the calculated formation enthalpies. The crystal structure information of these compounds are Cu (cubic, Fm−3m), Zn (hexagonal, P63/mmc), Sn (cubic, Fd−3m), S/Se (trigonal, P3121), CuS/Se (hexagonal, P63/mmc), Cu_2_S/Se (Monoclinic, P21/c), ZnS/Se (Zinc blende, F$\bar{43}$m), SnS/Se (orthorhombic, Pnma), SnSe2 (trigonal, P−$\bar{3}$M1) and Cu_2_Sn(S/Se)_3_ (monoclinic, Cc), CuSb(S/Se)_2_ (orthorhombic, Pnma)

**Table S1:** Calculated Formation enthalpies for CZTS/Se**.**

|  | **Present work** | **Reported work** | **Exp**^3^ |
| --- | --- | --- | --- |
| **CuS/Se** | -0.52/-0.48 | -0.53^1^/-0.46^2^ | -0.55 |
| **Cu_2_S/Se** | -0.93/-0.81 | -0.941^1^/ -0.61^2^ | -0.82 |
| **ZnS/Se** | -1.94/-1.8 | -1.96^1^/-1.47^2^ | -2.14 |
| **SnS/Se** | -0.91/-0.95 | -0.84^1^/-0.91^2^ | -1.04 |
| **Sn(S/Se)_2_** | -1.33/-1.28 | -1.31^1^/-1.03 ^2^ | -1.45 |
| **Cu_2_Sn(S/Se)_3_** | -2.65/-2.56 | -2.64^1^ /-2.11^2^ | -- |
| **CuSb(S/Se)2** | -1.27/-1.24 | -1.3^1^/-- | -- |

**Table S2:** The chemical potential values for CZTS/Se at the A–D points labeled in Fig. 2(a), 2(b) in units of eV.

|  | $\boldsymbol{\Delta}\boldsymbol{\mu}_{\mathbf{Cu}}$ | $\boldsymbol{\Delta}\boldsymbol{\mu}_{\mathbf{Zn}}$ | $\boldsymbol{\Delta}\boldsymbol{\mu}_{\mathbf{Sn}}$ | $\boldsymbol{\Delta}\boldsymbol{\mu}_{\mathbf{Sb}}$ |
| --- | --- | --- | --- | --- |
| **A** | -0.5/-0.5 | -1.55/-1.44 | -0.69/-0.58 | 0/0 |
| **B** | -0.5/-0.5 | -1.69/-1.43 | -0.56/-0.59 | 0/0 |
| **C** | -0.5/-0.5 | -2.12/-1.94 | -1.59/-1.55 | -0.73/-0.73 |
| **D** | -0.5/-0.5 | -1.92/-1.80 | -1.79/-1.69 | -0.73/-0.73 |

**Table S3:** Calculated Formation energy, Bulk modulus, Se vacancy Formation energy for CZTSe. Sn-Se means single Se is removed from the site nearest to Sn and Zn-Se means single Se is removed from the site nearest to Zn. The naming follows in the same manners.

| **System** | **Formation energy (eV)** | **Bulk modulus (GPa)** | **Vacancy Formation energy (eV)** |
| --- | --- | --- | --- |
| **CZTSe** | -- | 53 | Sn-Se = 1.68  Zn-Se = 1.65 |
| **Sb doped CZTSe** | 0.41 | 51.25 | Sb-Se = 1.26  Zn-Se = 1.65 |
| **Al doped CZTSe** | 0.95 | 52.83 | Al-Se = 2.1  Sn-Se = 1.66 |
| **Ga doped CZTSe** | 1.71 | 48.83 | Ga-Se = 1.58  Sn-Se = 1.66 |
| **Ba doped CZTSe** | -0.26 | 49.23 | Ba-Se = 0.36  Sn-Se = 1.37 |

**Bulk Modulus Calculation:**

The third order birch murnaghan equation of state is used to compute the bulk modulus through fitting the total energies for pure and dopes CZTS/Se by using following equation.

$$E_{s}=E_{0}+\frac{B_{0}V}{K(K-1)}\left[ K\left( 1-\frac{V_{0}}{V} \right)+\left( \frac{V_{0}}{V} \right)^{K}-1 \right]$$

Where B_0_, E_0_, V_0_, K are the fitting parameters (Bulk modulus, trail minimum energy, ground state total volume and first derivative of Bulk modulus).

**Figure S1:** Total energy plot as a function of volume by maintaining constant c/a ratio of (a) pure and (b-m) all doped CZTS systems. The red color lines represent the curve fitted using BM equation. All values are in eV.

**
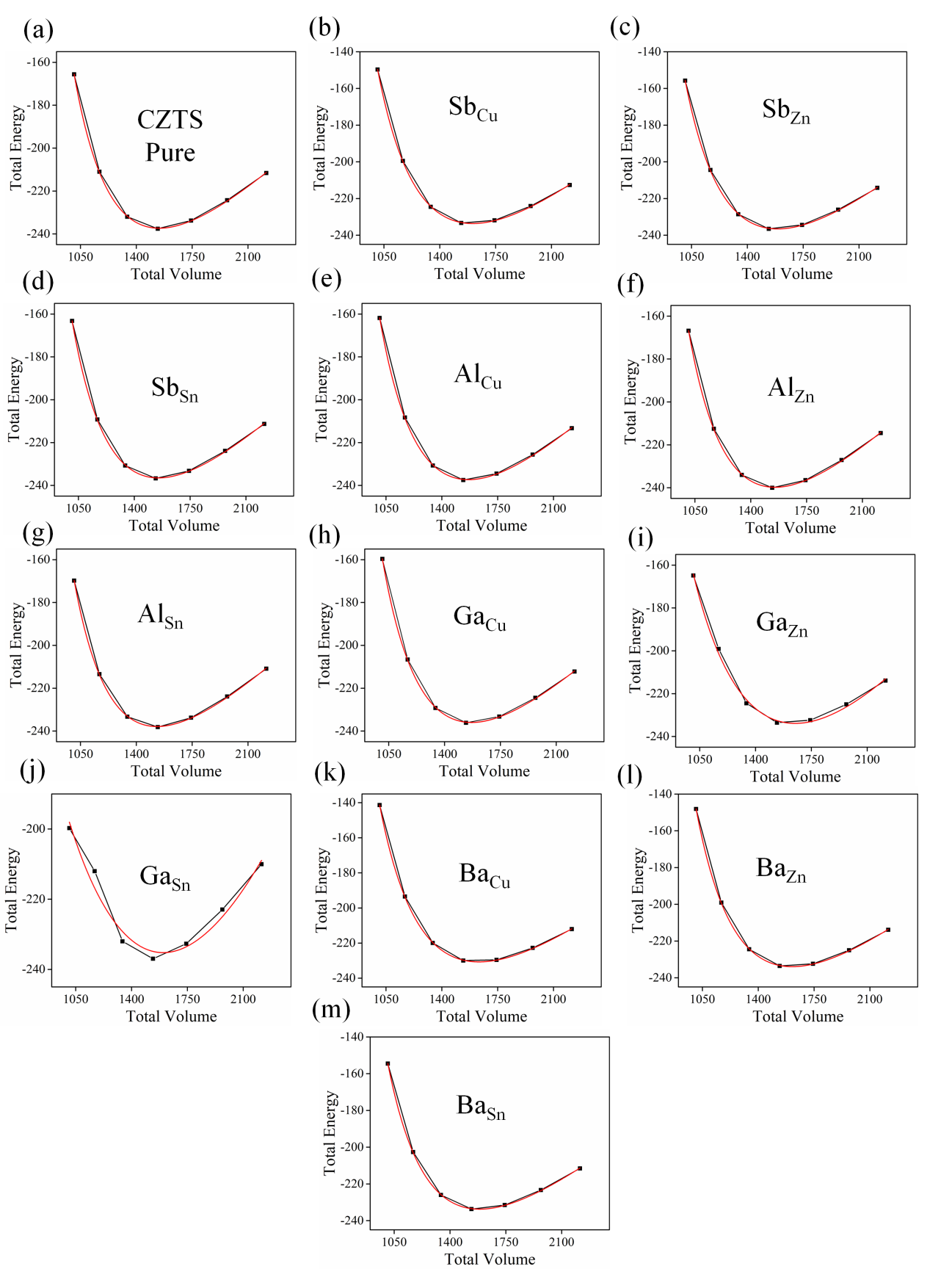
**

**Figure S2:** Total energy plot as a function of volume by maintaining constant c/a ratio of (a) pure and (b-m) all doped CZTSe systems. The red color lines represent the curve fitted using BM equation. All values are in eV.

**
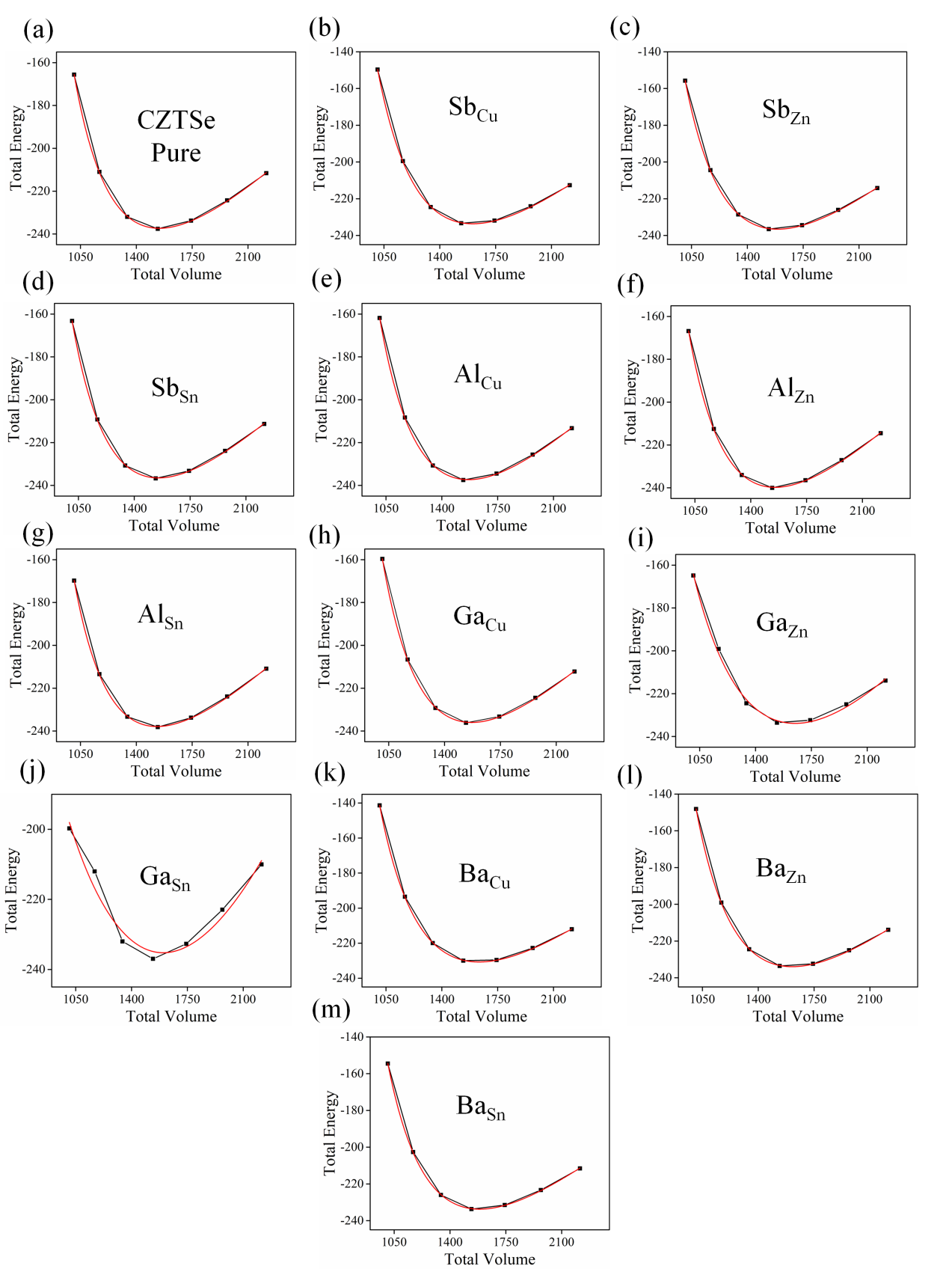
**

**Table S4:** The Bulk modulus value for Pure and doped CZTS/CZTSe

| **System** | **Bulkmodulus** |
| --- | --- |
| Pure | 65.80/53.00 |
| Sb_Cu_ | 60.00/50.23 |
| Sb_Zn_ | 62.29/50.65 |
| Sb_Sn_ | 65.00/51.25 |
| Al_Cu_ | 64.05/51.93 |
| Al_Zn_ | 65.69/52.83 |
| Al_Sn_ | 68.05/53.64 |
| Ga_Cu_ | 63.69/51.81 |
| Ga_Zn_ | 65.39/48.83 |
| Ga_Sn_ | 62.94/42.9 |
| Ba_Cu_ | 53.23/48.23 |
| Ba_Zn_ | 58.68/49.23 |
| Ba_Sn_ | 56.28/50.39 |

**Figure S3**: (a) calculated PDOS for pure CZTS (b) Sb doped CZTS (c) band structure for Sb doped CZTS.


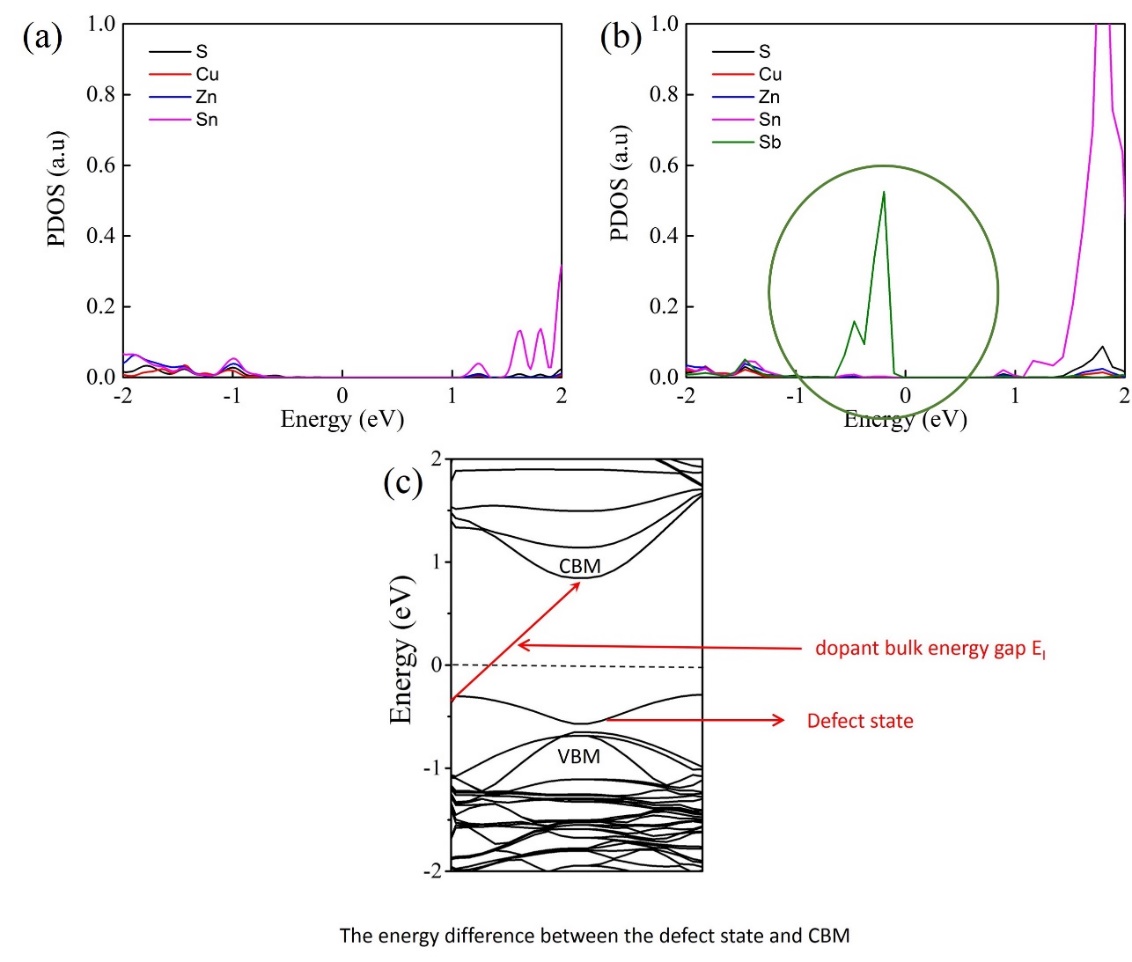


**Figure S4**: (a) calculated PDOS for pure CZTS (b) Al doped CZTS (c) band structure for Al/Ga doped CZTS.


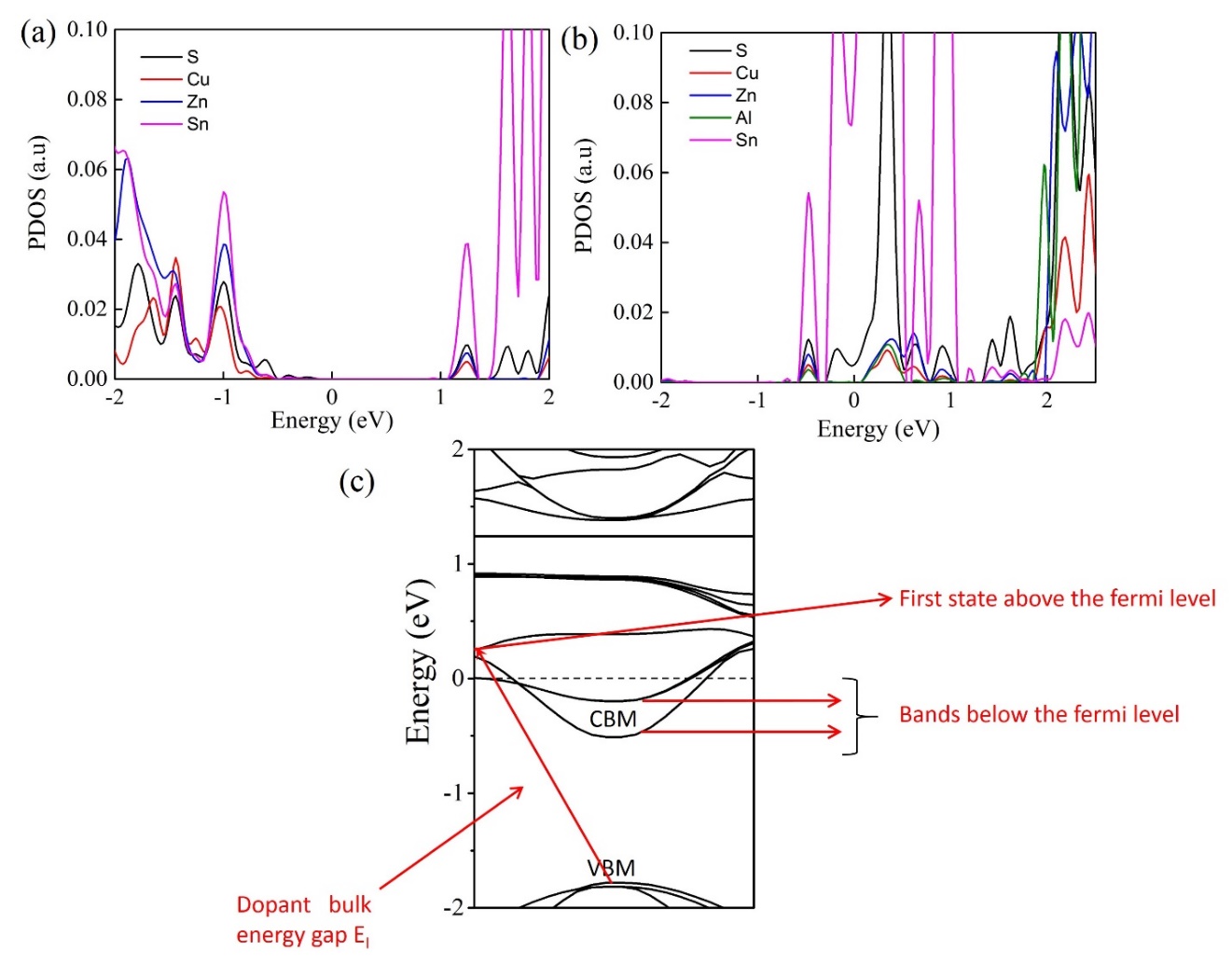


**Figure S5:** The calculated polyhedron of the stable chemical potential region of CZTS/Se **
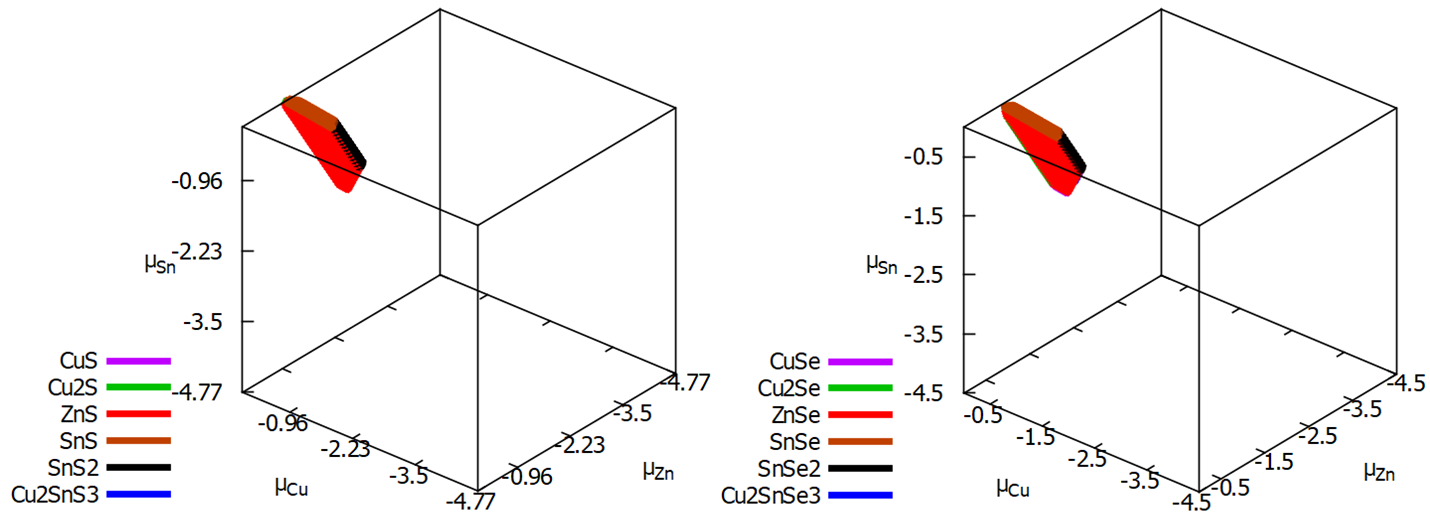
**

**Figure S6:** The calculated stable region of phase diagram for CZTS and CZTSe

as a function of Zn and Sn chemical potential with μ_Cu_ = 0 eV.

**
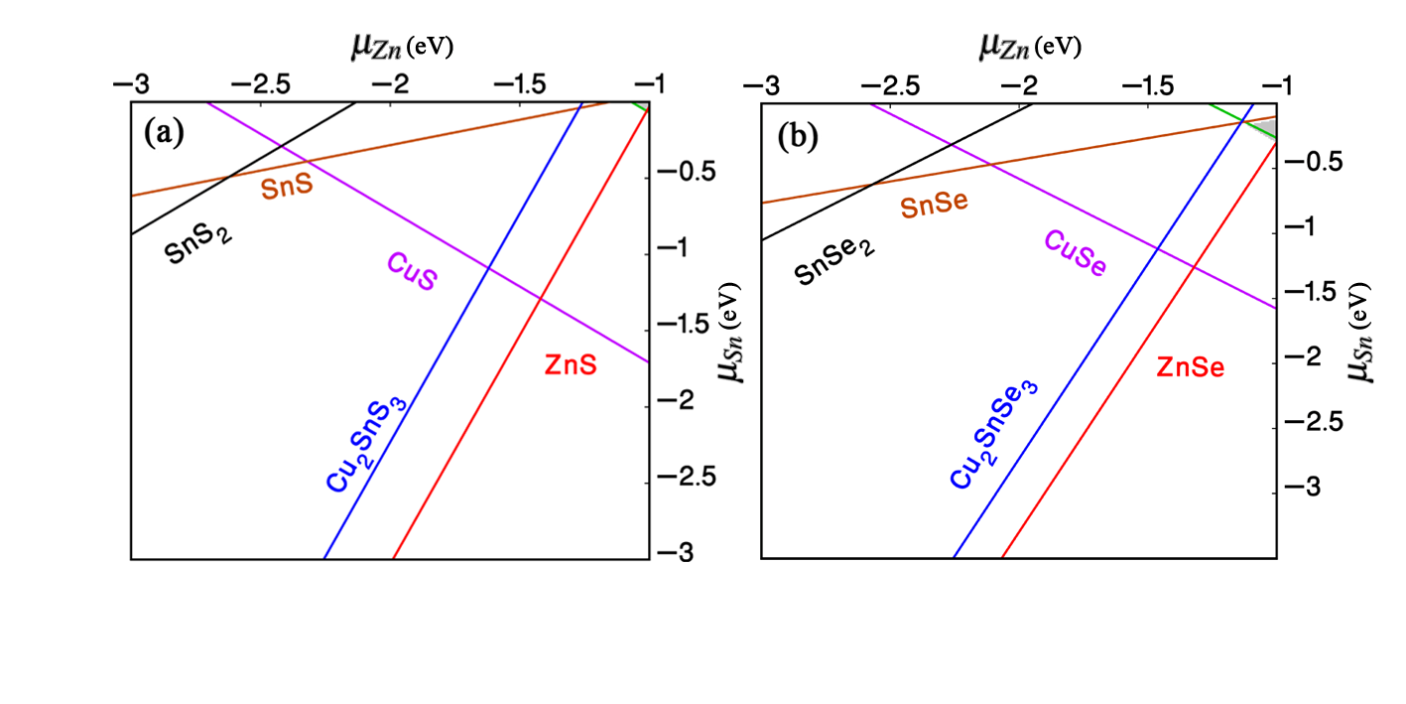
**

**Figure S7: (a) & (b)** The change of the defect formation energy in sb-doped CZTS and CZTSe

**
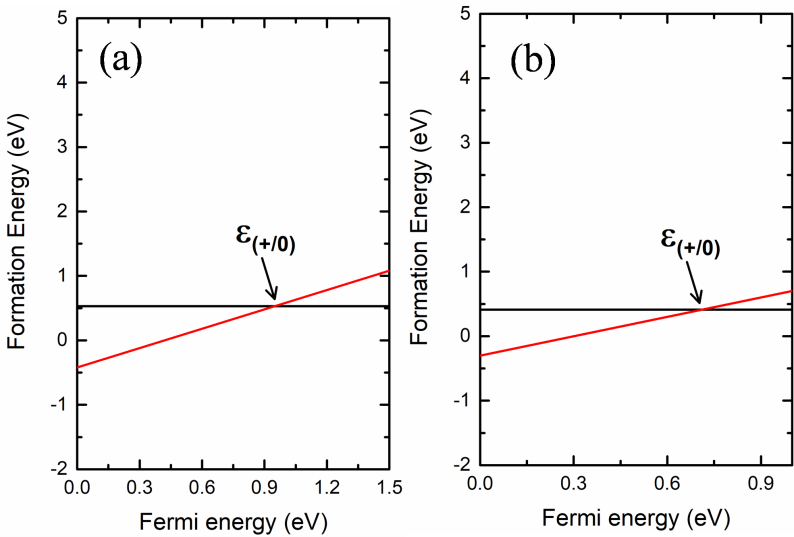
**

**Table S5:** Calculated bulk energy band gap, current density and upper limit of the energy conversion efficiency P (%) of pure and doped CZTS/Se considering 50nm film thickness.

| **System** | **Bulk-energy band gap (eV)** | **Thickness**  **(nm)** | **Current Density (mA/cm^2^)** | **P (%)** |
| --- | --- | --- | --- | --- |
| **Pure** | 1.43/0.85 | 50/50 | 20.01/35.08 | 12.12/25.87 |
| **Sb(Sn)** | 1.43/0.85 | 50/50 | 23.1/39.0 | 15.22/28.92 |
| **Al(Zn)** | 1.43/0.85 | 50/50 | 17.9/30.08 | 10.64/20.11 |
| **Ga(Zn)** | 1.43/0.85 | 50/50 | 18.2/31.4 | 10.87/20.51 |
| **Ba(Zn)** | 1.43/0.85 | 50/50 | 19.9/37.0 | 12.43/27.16 |
| **Sb+Al** | 1.43/0.85 | 50/50 | 22.8/39.4 | 14.82/29.07 |
| **Sb+Ga** | 1.43/0.85 | 50/50 | 23.2/39.9 | 15.15/29.53 |
| **Sb+Ba** | 1.43/0.85 | 50/50 | 23.2/40.7 | 15.16/34.69 |

**Figure S8:** (a)-(e) Demonstrate the calculated Absorbance and Photon Flux of CZTS under AM 1.5 G.


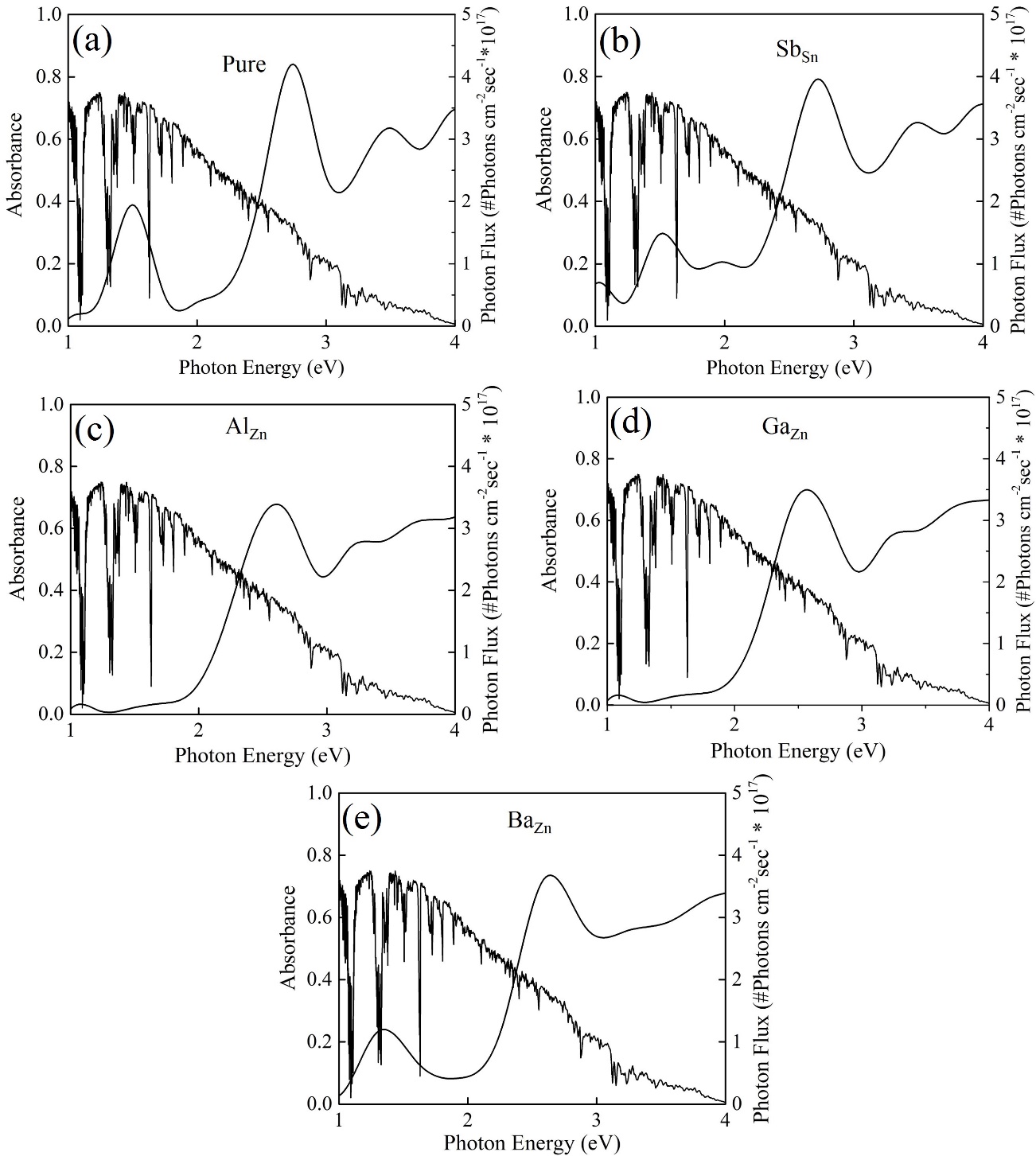


**Figure S9:** (a)-(e) Demonstrate the calculated Absorbance and Photon Flux of CZTSe under AM 1.5 G.


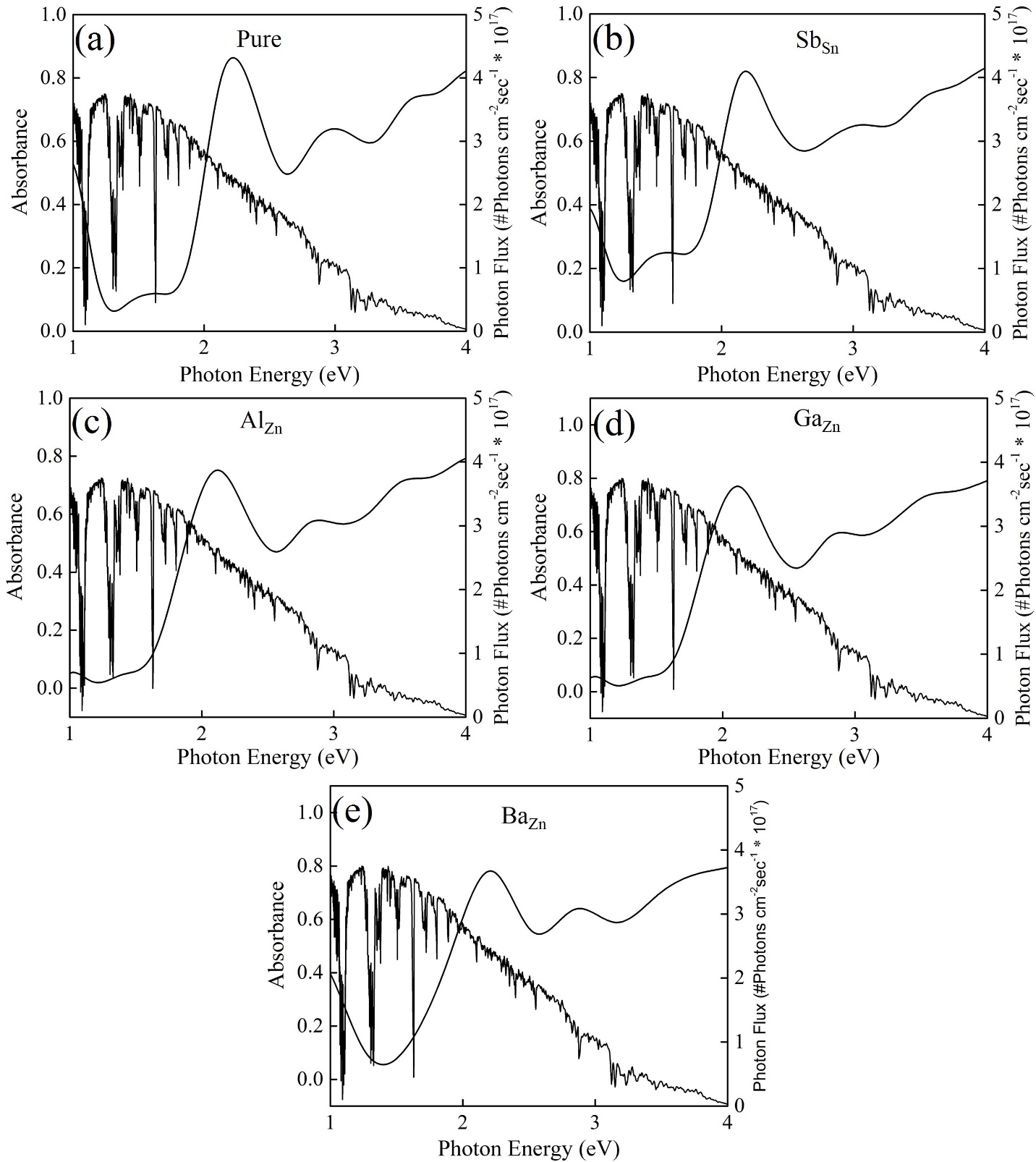


**Table S6:** Integral value of absorbance in the UV, IR, Visible range for pure and doped CZTS/Se. A(λ) is absorbance and the range 3.26 eV means 380 nm, 1.5 eV means 780 nm.

| **System** | **Visible ~** $\int_{\boldsymbol{1.5}}^{\boldsymbol{3.26}} \boldsymbol{A}\left( \boldsymbol{\lambda} \right)\boldsymbol{dE}$ | | **IR ~**$\int_{\boldsymbol{0}}^{\boldsymbol{1.5}} \boldsymbol{A}\left( \boldsymbol{\lambda} \right)\boldsymbol{dE}$ | |
| --- | --- | --- | --- | --- |
|  | **CZTS** | **CZTSe** | **CZTS** | **CZTSe** |
| **Pure** | 62.79 | 94.77 | 7.92 | 21.92 |
| **Sb(Sn)** | 70.5 | 102.92 | 9.35 | 23.67 |
| **Al(Zn)** | 58.56 | 90.94 | 0.77 | 1.56 |
| **Ga(Zn)** | 60.03 | 93.71 | 0.88 | 1.7 |
| **Ba(Zn)** | 63.2 | 91.49 | 8.7 | 21 |

**Figure S10**: The band structures of the pure CZTS and CZTSe and the Sb_Sn_, Al_Zn_, Ga_Zn_, Ba_Zn_ doped CZTS/Se, top layer shows for CZTS and bottom layer shows for CZTSe by using PBE+U correction method.


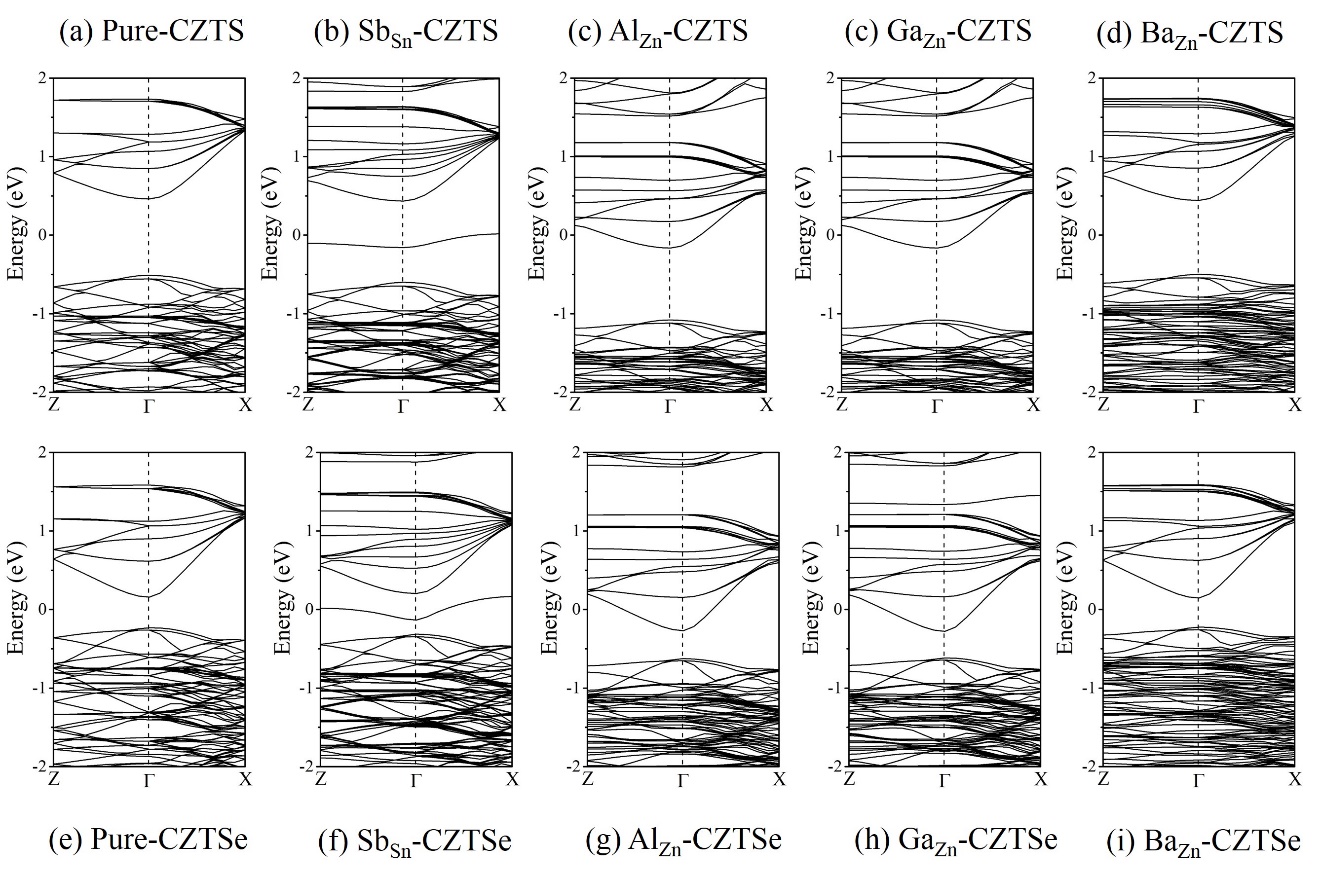


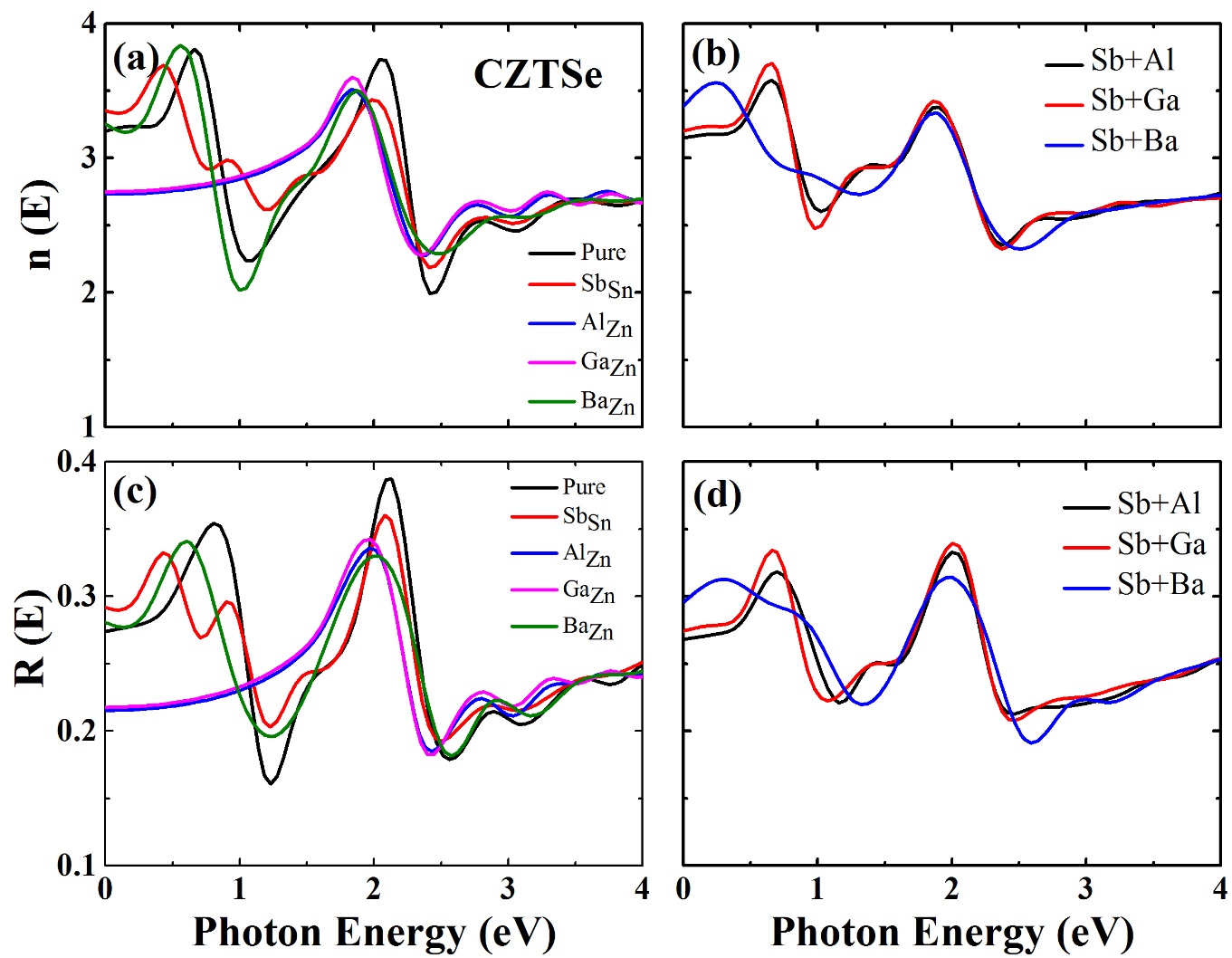


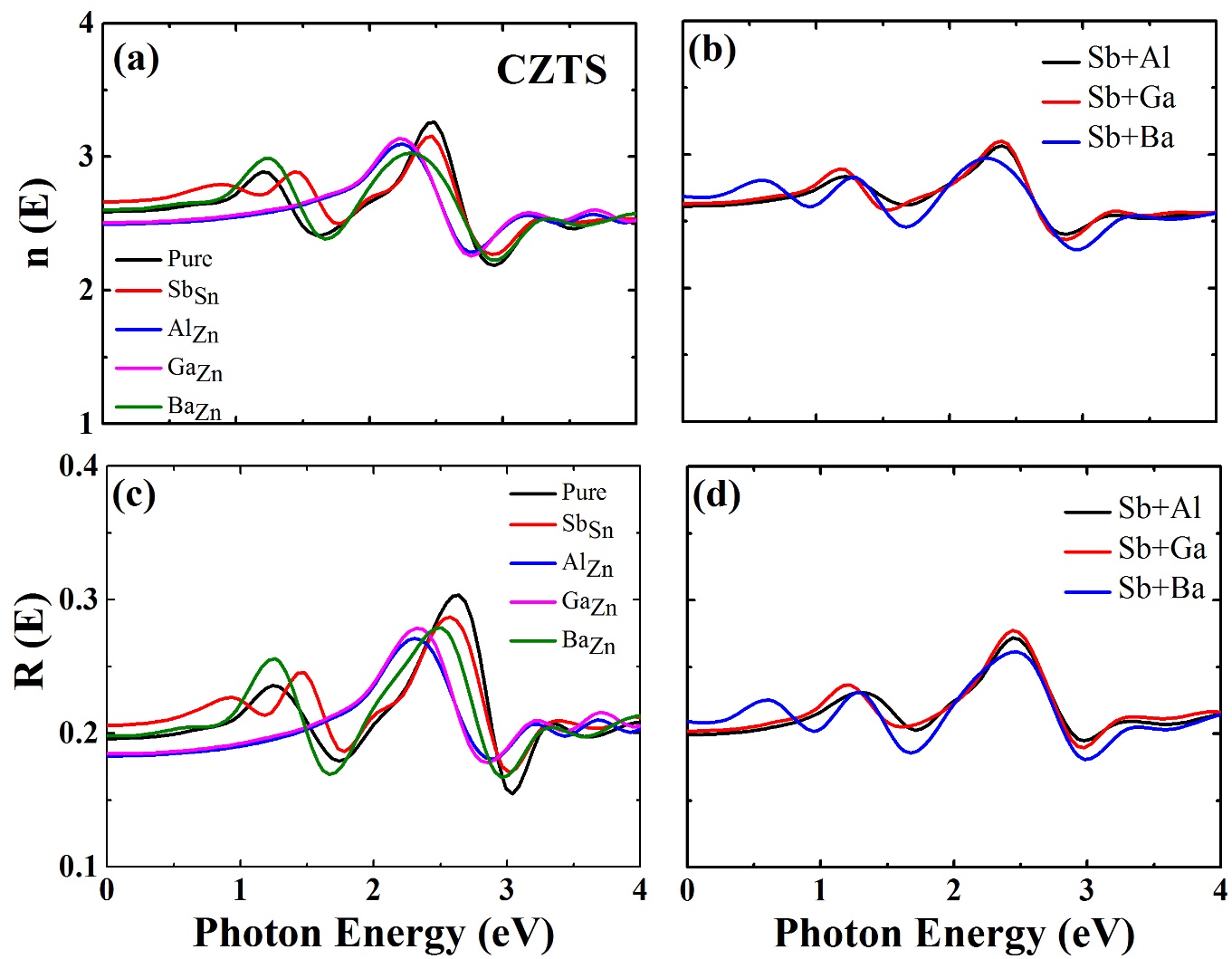
**Figure S11:** Calculated (a) Refractive index of pure, Sb_Sn_, Al_Zn_, Ga_Zn_, Ba_Zn,_ CZTSe (b) Refractive index of Sb+Al, Sb+Ga, Sb+Ba CZTSe (c) Reflectivity of pure, Sb_Sn_, Al_Zn_, Ga_Zn_, Ba_Zn,_ CZTSe (d) Reflectivity of Sb+Al, Sb+Ga, Sb+Ba CZTSe

**Figure S12:** Calculated (a) Refractive index of pure, Sb_Sn_, Al_Zn_, Ga_Zn_, Ba_Zn,_ CZTS (b) Refractive index of Sb+Al, Sb+Ga, Sb+Ba CZTS (c) Reflectivity of pure, Sb_Sn_, Al_Zn_, Ga_Zn_, Ba_Zn,_ CZTS (d) Reflectivity of Sb+Al, Sb+Ga, Sb+Ba CZTS

**References:**

1. X. Zhang, M. Han, Z. Zeng and Y. Duan, The role of Sb in solar cell material Cu_2_ZnSnS_4_, J. Mater. Chem. A, **5**, 6606 (2017).
2. Chaochao Dun, N. A. W. Holzwarth, Yuan Li, Wenxiao Huang, and David L. Carroll, Cu_2_ZnSnSxO_4-x_ and Cu_2_ZnSnS_x_Se_4-x_: First principles simulations of optimal alloy configurations and their energies, J. Appl. Phys. **115**, 193513 (2014)
3. D. R. Lide, CRC Handbook of Chemistry and Physics, 84th ed. (CRC, Boca Raton, 2003).
